# Review of In-Pixel Gain Amplifiers and Readout Circuits for Low-Light CMOS Image Sensors

**DOI:** 10.1109/sr.2025.3560917

**Published:** 2025-04-14

**Authors:** AMR M. MAGHRABY, IBRAHIM BOZYEL, ISLAM T. ABOUGINDIA, SUAT U. AY

**Affiliations:** 1 Electrical and Computer Engineering Department, University of Idaho, Moscow, ID 83843 USA; 2 Electrical and Computer Engineering Department, Worcester Polytechnique Institute, Worcester, MA 01609 USA; 3 Electronics Engineering Department, Military Technical College, Cairo 11371, Egypt

**Keywords:** Conversion gain (CG), in-pixel amplifiers, low-noise readout integrated circuit (ROIC), Readout integrated circuit (ROIC)

## Abstract

The low-light performance of image sensors can be enhanced by designing high-effective conversion gain signal paths from photon conversion sites to chip outputs. This can be achieved by using high-gain in-pixel amplifiers, high-gain column amplifiers, and by reducing the conversion capacitance of photon-sensing nodes. However, each of these approaches presents unique challenges and limitations that have restricted their widespread adoption in low-light applications. This article reviews the use of in-pixel gain amplifiers and their signal chain electronics in high-conversion-gain complementary metal–oxide–semiconductor (CMOS) image sensors over the past two decades. In-pixel gain amplifiers are classified into different categories according to the type of amplification technique used. Analyses of the column-referred conversion gain and the noise of each topology are presented alongside the different metrics used to characterize CMOS image sensor pixels for low-light imaging applications. The performance metrics of various in-pixel gain amplifiers are compared, providing a framework that highlights the best achieved input-referred noise in CMOS image sensors over the past 15 years. Furthermore, different tradeoffs are examined between optimizing conversion gain, pixel full-well capacity, and input read noise in both voltage and charge domains.

## I. INTRODUCTION

During the past two decades, complementary metal–oxide–semiconductor (CMOS) image sensors (CIS) have dominated the image sensor market, gradually taking over the market share of the incumbent charge-coupled device (CCD) image sensors. Integrating imaging elements along with the sensor electronics and signal processing capabilities on a single chip allowed this transition. Furthermore, CIS emerged as the preferred imaging technology due to its reduced size and power consumption, driven by the scaling of CMOS technology nodes in accordance with Moore's law. This scaling trend fueled the integration of more and more intelligence on-chip, allowing system-on-a-chip CIS cameras on many systems, such as laptops, cell phones, cars, surveillance, medical imaging, and professional cameras. Besides, CIS offers lower manufacturing costs in contrast to CCD [1].

Imaging in low-light environments is essential in many applications, such as science, military surveillance systems, space exploration, and security. Therefore, there is an increased need for CIS cameras that possess high sensitivity and can effectively operate in low-light environments. The read noise of the CIS signal chain plays a crucial role in these imaging systems. Consequently, it limits the eventual detection performance of such imaging systems [2]. The read noise is measured at the output of the CIS and commonly referred back to the input of the signal chain as the minimum root-mean-squared (rms) number of detectable electrons (e-rms) on the sensing junction of the pixel [3]. In recent years, the terms subelectron and deep subelectron read noise were proposed by researchers referring to sub-1e-rms read noise of CIS signal chains [2], [4], [5], [6], [7], [8]. This is necessitated by the use of the pinned photodiode and back-side illumination (BSI) that enhanced the sensitivity of CIS by reducing the dark current and the pixel electronics noise [9].

A variety of approaches was proposed to reduce the read noise of CIS. High-gain column-level amplifiers have been extensively employed as a solution in low-light level applications due to their notable efficiency in reducing input-referred noise [10], [11], [12]. Alternatively, the reduction of the sensing node's capacitance results in a high conversion gain (CG) pixel, which in turn leads to a lower input-referred noise electrons [6], [9], [13], [14], [15]. In addition, correlated double sampling (CDS), correlated multiple sampling (CMS), and other noise shaping techniques in analog and/or digital domains pushed the read noise of the pixels to fundamental limits [11], [16], [17], [18], [19], [20]. Besides, a high amplification factor at the very beginning of any readout signal chain is indeed very effective in reducing the input-referred noise generated by the readout circuits. However, most CIS pixels proposed in the literature use a source follower (SF) to buffer the photogenerated and converted voltage signals from the photosensitive elements. This is mainly due to their high linearity over reasonable input and output voltage ranges. Several techniques have been used for increasing the gain linearity and range of SF-based pixels, including boosting pixel control signals [21], [22], using buried-channel transistors [23], and using bulk-tied positive channel metal oxide semiconductor fiel effect transistor (PMOS) transistors [10], [23], [24]. Most notably, open-loop in-pixel gain amplifier (IPGA) topologies were used to actively amplify pixel signals instead of pixel source followers (PSFs) with close to unity gain [2], [7], [25].

There are two types of IPGAs with voltage gain greater than unity: open-loop amplifiers [2], [7], [25] and closed-loop amplifiers [8], [26], [27]. One of the metrics used to characterize a CIS is the column-referred conversion gain (CCG) [28]. This metric results from multiplying the floating diffusion CG by the IPGA's gain. In order to compare various IPGA topologies, the CCG of various architectures will be computed whenever data are available.

This review aims to provide an overview of the progress of in-pixel CIS gain amplifiers and their realizations in the past few decades. The rest of this article is organized as follows. Section II discusses and categorizes various IPGA topologies, highlighting their voltage gain characteristics and associated noise. Section III evaluates and compares the performance and noise characteristics of different IPGA topologies against several CIS metrics. Section IV explores emerging trends and future directions for IPGAs in next-generation CIS. Finally, Section V concludes this article.

## II. IPGA TOPOLOGIES

Over the years, different CMOS amplifiers with small-signal gain larger than unity were proposed for pixel-level amplification of photogenerated signals. However, PSF remains the mainstream in-pixel amplifier used in CIS nowadays. In this section, different IPGAs are reviewed along with the PSF, which is reviewed as a reference for the presented IPGA topologies.

### A. PSF AMPLIFIER

The P-type common-drain (CD) amplifier, also known as SF, is considered the first active pixel amplifier used in MOS image sensors in 1968 by Noble [29] and Chamberlain [30]. It becomes the main and only active electronics in CCD image sensors buffering transferred and converted pixel charges in global amplifiers at the end of the readout signal chain [31]. When CIS was reintroduced in early 1990s [32], [33], [34], [35], SF was also the choice of the in-pixel amplifier to buffer pixel signals to column readout electronics that amplify the signals with programmable gain and reduce the noise generated mainly by the PSF. Since PSF have less than unity gain, they can attenuate both signal and pixel noise equally, reducing output-referred noise.

**FIGURE 1.**
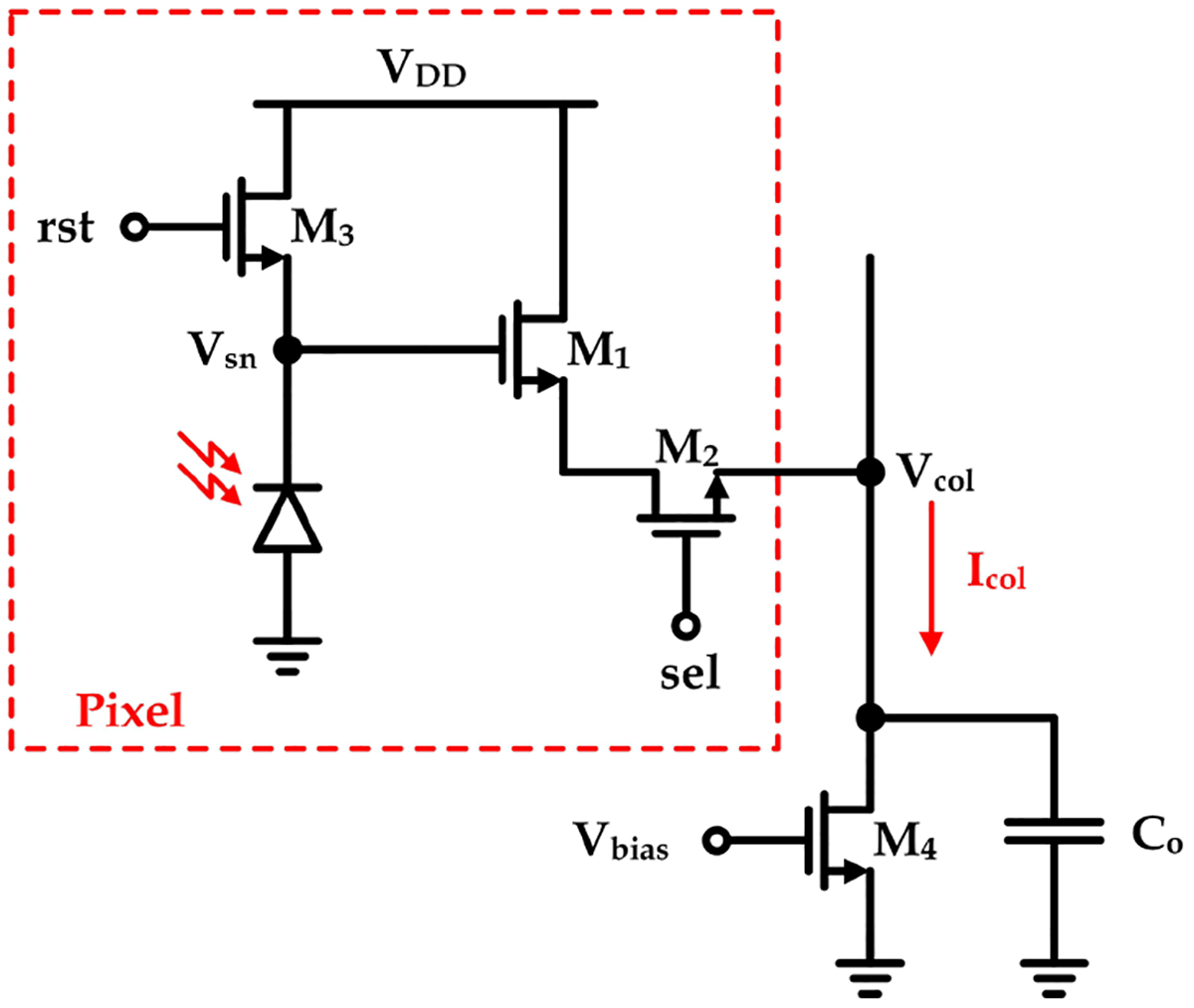
PSF amplifier in a CIS.

Fig. 1 shows the schematic of a typical PSF amplifier integrated into a CIS pixel. M1 acts as the in-pixel SF transistor, while M2 and M3 are the select and reset transistors. When accessed ($\text{sel}=V_{DD}$), PSF transistor buffers the sense node (SN) voltage ($V_{\text{sn}}$) to column electronics ($V_{\text{col}}$). An active load transistor M4 is placed on the column side to provide bias current ($I_{\text{col}}$) to the PSF transistor. Although a standard SF amplifier has a gain factor of about 0.8 [36], it is considered to be the most linear single-stage amplifier to be used for buffering SN signals in CIS. The voltage gain of the PSF in Fig. 1 can be found by assuming that M2 works in the linear region and by ignoring body transconductance as presented in the following equation:

(1) $$A_{\text{SF}}=\frac{V_{\text{col}}}{V_{\text{sn}}}=\frac{g_{m1}r_{o4}}{1+\left(1+\frac{R_{ds2}}{r_{o4}}\right)g_{m1}r_{o4}}$$

where $R_{ds2}$ is the linear on resistance of M2, and $r_{o4}$ is the output resistance of transistor M4. Since M2 works in the linear region, its on resistance changes with the output voltage, as captured by the following equation:

(2) $$R_{ds2}=\frac{1}{\beta_{2}\left(V_{DD}-V_{\text{out}}-V_{\text{th}2}\right)}.$$

Assuming ($g_{m1}r_{o4}>1$) and ($r_{o4}>R_{ds2}$), gain equation simplifies to

(3) $$A_{\text{SF}}=\frac{V_{\text{col}}}{V_{\text{sn}}}≅\frac{1}{\left(1+\frac{R_{ds2}}{r_{o4}}\right)}$$

which is less than unity and gets smaller if $V_{\text{out}}$ becomes smaller. In column electronics, the PSF gain can be enhanced by increasing $r_{o4}$ through a longer channel length for M4 or by raising the dc $I_{\text{col}}$ current. Further linearization and improvement of the gain are possible by boosting the driving voltage of the sel signal beyond $V_{DD}$ effectively reducing $R_{ds2}$ of M2 [22].

The CCG defines the relationship between the generated voltage at the pixel output and the number of photoelectrons converted at the SN. It is given by

(4) $$\text{CCG}=\frac{V_{\text{col}}}{Q_{\text{sn}}}=\frac{A_{\text{SF}}}{C_{\text{Sn}}+C_{gd1}+C_{gs1}\left(1-A_{\text{SF}}\right)+C_{gs3}}.$$

The presented CCG in (4) assumes a low-frequency operation. Therefore, to increase the CCG of the pixel, the SF gain has to be maximized. This will ensure the cancellation of the gate-to-source capacitance of the M1. Further increase of CCG can be achieved by reducing the SN capacitance ($C_{\text{sn}}$) and the other overlap capacitances of M1 and M3.

The noise model and output-referred noise of SF-based CIS pixel were presented by Tian et al. [37] for ideal small-signal transistor models. A more comprehensive representation of the band-limited wideband noise of the PSF transistors, referred to the SN, can be derived as follows:

(5) $${\overset{‾}{v}}_{ni}^{2}=\frac{kT\gamma }{C_{o}}\cdot \left(\frac{1}{A_{\text{SF}}}\right)\cdot \left(\frac{1}{A_{\text{is}}}+\frac{1}{1+\frac{1}{A_{\text{is}}-1}}+\frac{g_{m4}}{g_{m1}}\cdot A_{\text{is}}\right).$$

Here, $A_{\text{SF}}$ is the PSF gain presented in (3), while $A_{is}$ is the intrinsic gain factor of M1 and M2 given as $\left(1+g_{m1}R_{ds2}\right)$. Equation (5) assumes that $g_{m1}r_{o1}>1,g_{m1}r_{o4}>1$, and M2 transistor works in the linear region with $R_{ds2}$ given in (2). The terms in the right parenthesis of (5) are the input-referred noise contributions of the PSF transistors M1, M2, and M4, respectively. From (5), it can be seen that a typical PSF gain of 0.8 causes an increase in the input noise. Improving select transistor's linear on resistance (i.e., by boosting [22], [22]) would improve the $A_{is}$ and reduces the input-referred noise contribution of M1 and M2. However, this improvement has to be balanced by reducing the transconductance of M4 $\left(g_{m4}\right)$ to reduce M4 contribution to the input noise.

### B. $G_{m}$-C-BASED PIXEL AMPLIFIERS

**FIGURE 2.**
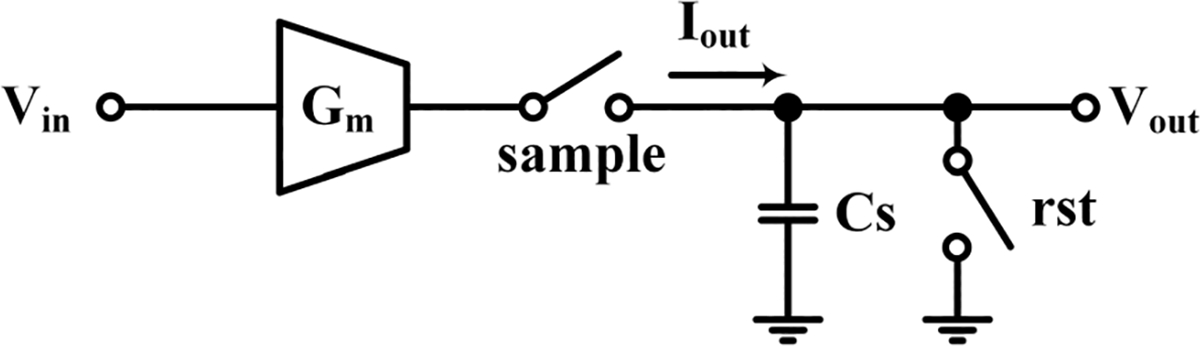
$G_{m}$-C-based amplifier.

A $G_{m}$-C-based amplifier consists of a transconductor $\left(G_{m}\right)$, which converts an input voltage into an output current, and an integration capacitor ($C_{S}$), which converts transconductor current into voltage, as shown in Fig. 2. In the CIS setup, the pixel SN converts photogenerated charges into a voltage that serves as the input to the transconductor. This pixel voltage is then converted into current, which is subsequently integrated on a column capacitor to produce the output voltage. This configuration is commonly referred to as an OTA-C or $G_{m}\text{-C}$ integrator. The corresponding equation describing its operation is given as

(6) $$V_{\text{out}}(t)=\frac{1}{C_{s}}\int_{0}^{T_{\text{int}}}I_{\text{out}}(t)\cdot dt=\frac{G_{m}}{C_{s}}\int_{0}^{T_{\text{int}}}V_{\text{in}}(t)\cdot dt$$

where $T_{\text{int}}$ is the integration time over the capacitor ($C_{s}$). The gain of the circuit can be found to be

(7) $$A_{v}=\frac{V_{\text{out}}}{V_{\text{in}}}=\frac{G_{m}}{C_{s}}\cdot T_{\text{int}}.$$

**FIGURE 3.**
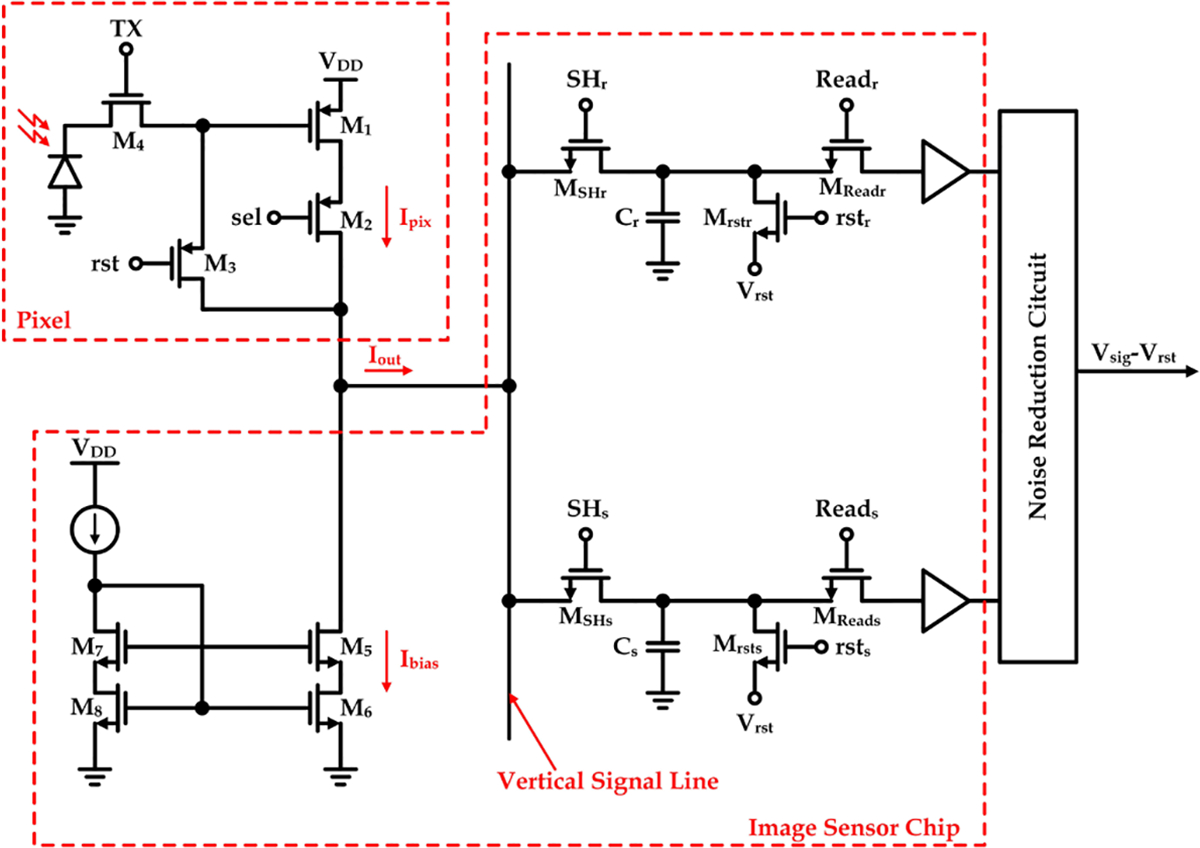
Charge-mode $G_{m}$-cell-based pixel amplifier proposed by Ge and Theuwissen [2], including column bias and processing circuits.

One of the notable $G_{m}$-C-based amplifier for CIS was presented by Ge and Theuwissen [2], the so-called charge-mode readout $G_{m}$-cell-based pixel amplifier with an adjustable CCG. The proposed architecture, as illustrated in Fig. 3, employs an in-pixel transconductor and a shared column capacitor facilitating integration. Unlike fixed-gain, voltagemode in-pixel common-source (CS) amplifiers that constrain the CIS pixel's dynamic range, this design allows for a tunable CCG by modifying the charging time, thereby enhancing the sensor's dynamic range. The charging time can be adjusted between 100 ns and 4μs, resulting in a CCG ranging from 50 to $1600\;\mu \text{V}/{\text{e}}^{-}$. The in-pixel voltage gain varies from 1 to 32 V/V, while the SN's CG (CG_SN_) is $55\;\mu \text{V}/{\text{e}}^{-}$. In addition, the $G_{m}$-cell-based configuration offers a noise advantage by forming a continuous-time first-order Sinc-type low-pass filter, as opposed to the first-order low-pass filter typically inherent to voltage mode pixel amplifiers. This improved filtering enhances the noise performance of $G_{m}\text{-C}$ pixels by 20% compared to PSF sensors.

The $G_{m}$-cell-based pixel utilizes three p-type mosfet transistors for readout: a CS transistor (M1), a select transistor (M2), and a reset transistor (M3). To maintain the required input–output linearity, the time constant of the $G_{m}\text{-C}$ integrator must be significantly larger than the charging time. To achieve this requirement, M2 is biased in saturation and functions as a cascode transistor, enhancing the output resistance of the $G_{m}$-cell-based pixel readout. In addition, using M2 as a cascode transistor helps mitigate the Miller effect [25].

The design incorporates two column-level charging capacitors for reset ($C_{r}$) and signal ($C_{s}$), each with a capacitance value of 2 pF. The $G_{m}$-cell-based pixel was designed with an output resistance of 200MΩ and a transconductance ($G_{m}$) of 30μS. A 60 × 64 pixel array was fabricated using 180-nm CMOS CIS process technology, featuring a pixel pitch of 11μm. To simplify the pixel layout, no in-pixel resistors or capacitors were used. Analog-to-digital conversion is performed off-chip using a 16-bit analog-to-digital converter (ADC), and CDS is performed digitally after conversion. The noise analysis of the $G_{m}$-cell-based pixel amplifier was presented in [3]. The following equation represents the input-referred noise due to thermal noise and it assumes that a charging time ($T_{\text{ch}}$) is much lower than the integrator's time constant:

(8) $${\overset{‾}{v}}_{\text{in},n_{\text{th}}}^{2}=\frac{4}{3}\cdot KT\left(\frac{1}{g_{m1}}+\frac{g_{m6}}{g_{m1}^{2}}\right)\cdot \frac{1}{T_{\text{ch}}}.$$

Han and Theuwissen [7] introduced a modified version of $G_{m}$-cell-based pixel in 2021, incorporating column current modulation to address two key issues: 1) charge injection errors caused by the auto-zero reset switch (M3), and 2) mismatches between $I_{\text{pix}}$ and $I_{\text{bias}}$ due to the differing temperature coefficients of transistors M1 and M6. In addition, a secondary infinite impulse response (IIR) filter can be used at the column level to enhance the CCG and reduce noise.

An off-chip controller regulates the column bias currents, ensuring that all transistors in both the pixel and column operate in the saturation region. This current compensation mechanism allows the charging time to be extended up to 14μs. As a result, the CCG increases to $2200\;\mu \text{V}/{\text{e}}^{-}$ at a 14-μs charging time, compared to $1600\;\mu \text{V}/{\text{e}}^{-}$ in [2].

**FIGURE 4.**
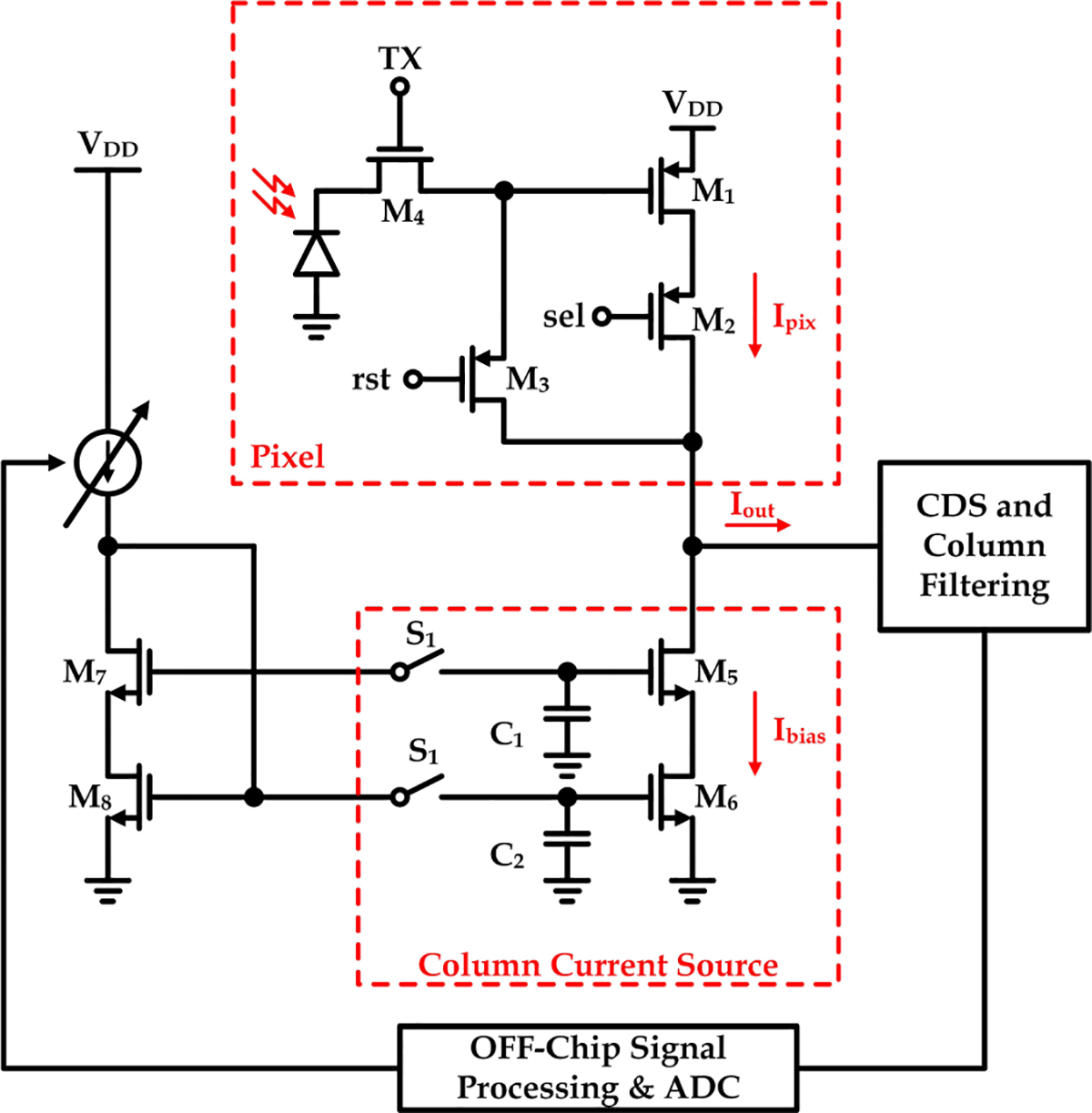
Modified $G_{m}$-cell-based pixel amplifier proposed by Han and Theuwissen [7].

The in-pixel transconductance amplifier achieves an input-referred noise of 0.31 e^−^ rms and employs a sampling capacitor of $C_{\text{FIR}}=3.4\;\text{pF}$. A 256 × 128 pixel array was fabricated using a 180-nm CMOS CIS process, with a pixel pitch of 10μm. Fig. 4 illustrates the schematic of the pixel architecture.

The gain of the $G_{m}$-cell-based pixel amplifier, extracted from the available data, is configurable from 9 V/V at a 1-μs charging time to 123 V/V at 14μs. At the 14-μs charging time, the peak noise was measured at 0.42 e^−^ rms, while the minimum noise reached 0.31 e^−^ rms. One of the main benefits of the $G_{m}\text{-C}$ structure introduced in [2] and [7] is its ability to be easily adjusted for imaging in both low- and high-exposure environments.

### C. $G_{m}$-R-BASED IN-PIXEL AMPLIFIERS

**FIGURE 5.**
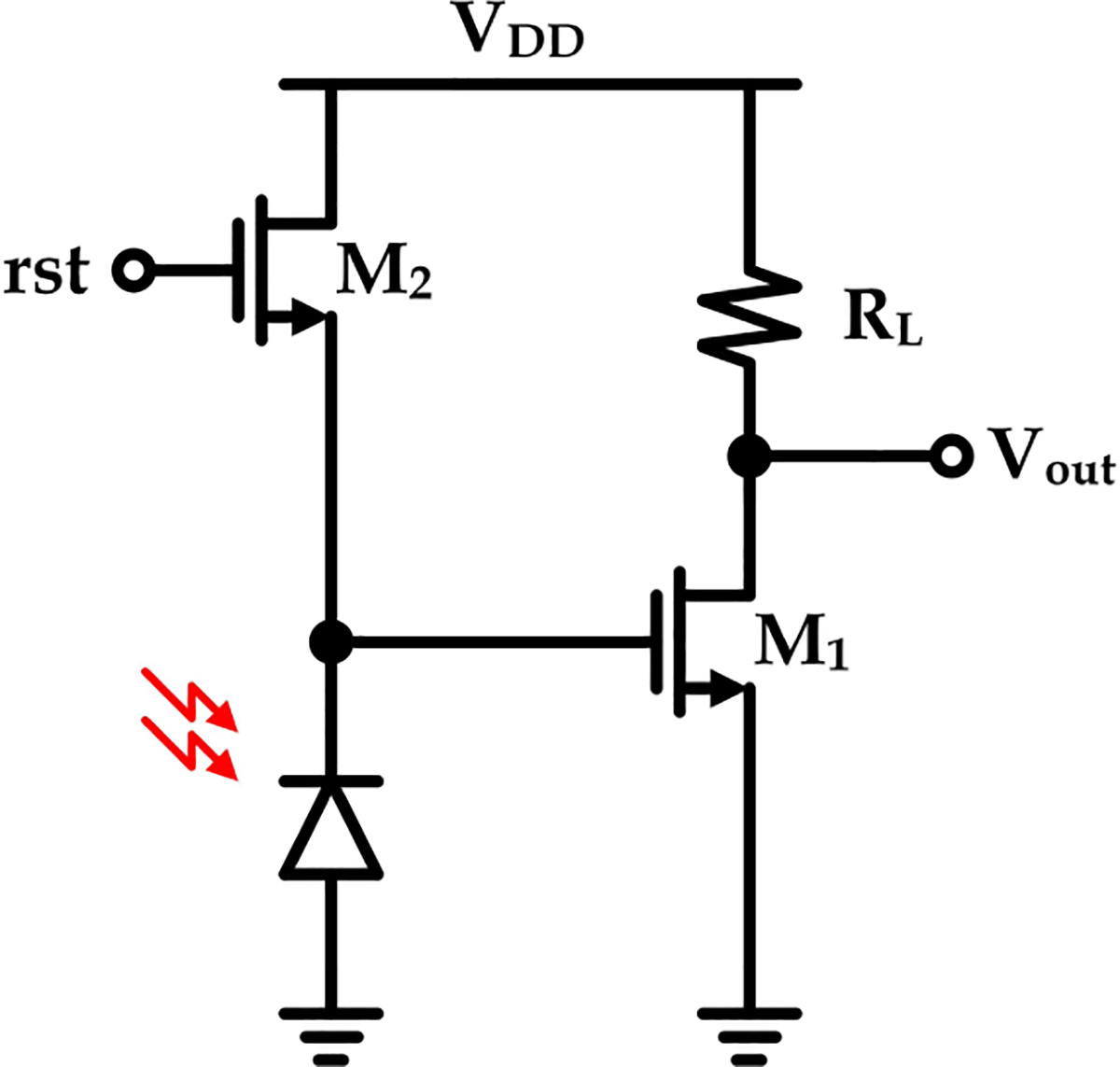
CS amplifier built-in the hybrid SOI CMOS APS technology in [38].

A $G_{m}$-R-based amplifier configuration consists of a transconductor cell that produces an output current based on its input voltage and a linear load resistor. One possible implementation of a $G_{m}\text{-R}$ configuration is a CS or cascode amplifier with a passive load resistor. The current generated by the $G_{m}$ cell results in a corresponding output voltage across the load resistor. To enhance the gain, two key factors must be considered: 1) increasing the transconductance $G_{m}$ of the cell, and 2) increasing the load resistance. The $G_{m}\text{-R}$ circuit is illustrated in Fig. 5, and its gain equation is given as follows:

(9) $$V_{\text{out}}=I_{\text{out}}\cdot R_{L}=G_{m}\cdot V_{\text{in}}\cdot R_{L}$$

(10) $$A_{v}=\frac{V_{\text{out}}}{V_{\text{in}}}=G_{m}\cdot R_{L}.$$

In 1999, Zhang et al. [38] suggested using a special process to etch the buried oxide of the silicon-on-insulator (SOI) wafer and grow the photodiode on the bottom substrate. The rest of the pixel is built on the top thin silicon film of the SOI wafer. This is called hybrid active pixel sensor (APS) on SOI CMOS process substrate [39]. The pixel used two different configurations, the CS and CD configurations. Fig. 5 shows the implementation of the CS configuration. Zheng et al. [40] proposed a similar structure with only SF as the amplifying device.

SOI CMOS technology suffers from the limited silicon substrate thickness and, hence, the reduced quantum efficiency as many photons dive deeper without being collected by the photodiode. However, it is advantageous to use SOI due to its radiation hardness with space or military applications. On the other hand, the bulk technology offers the higher quantum efficiency required by the image sensor.

The pixel pitch of [38] was 20μm, and the voltage gain of the SF was reported to be 0.75 V/V. However, no voltage gain or CG of the CS configuration was reported in the article.

**FIGURE 6.**
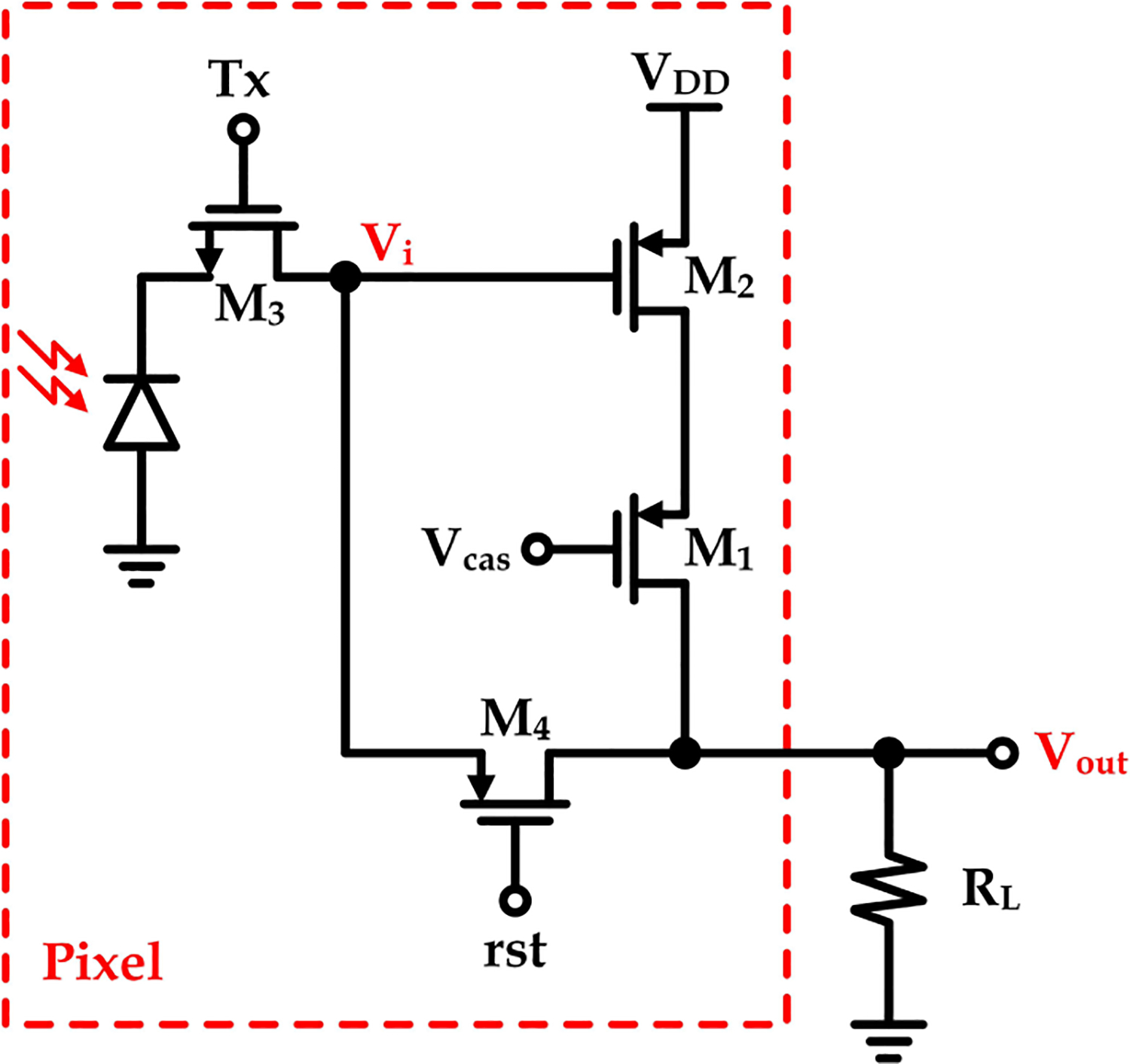
In-pixel CS amplifier as proposed by Lotto et al. [25].

Lotto et al. [25] proposed a Gm-R amplifier using an open-loop P-type cascode CS amplifier. The proposed architecture is illustrated in Fig. 6. It consists of a 4T-pixel electronics with a column-level resistance $R_{L}$. The pixel employs a self-reset transistor M4 in negative feedback, which sets the $G_{m}$ cell at its optimum bias point before transferring the photogenerated charge to the input of the $G_{m}\text{-R}$ cell. To mitigate the Miller effect, the pixel uses M1 as a cascode transistor as adopted in [2] and [7]. The open-loop voltage gain of the in-pixel amplifier was 10 V/V, which prevents the need for a low-pass filter in the signal readout chain. Due to the relatively high voltage gain, the exposure time for the sensor was limited to 50μs in dark conditions to generate an image.

An active pixel array of 256 by 256 was designed and fabricated in a 180-nm CMOS CIS process. The pixel pitch was reported to be 11μm. The gain of such an amplifier depends on the transconductance of $M_{2}$ and the load resistance $R_{L}$, as shown in the equation below. The amplifier design achieved a subelectron read noise of 0.86e- and a CCG of 300μV/e-

(11) $$A_{V_{t}}=-g_{m2}\left(g_{m1}r_{o1}r_{o2}\|R_{L}\right)\approx -g_{m2}R_{L}.$$

### D. IN-PIXEL CAPACITIVE TRANSIMPEDANCE AMPLIFIER (CTIA)

**FIGURE 7.**
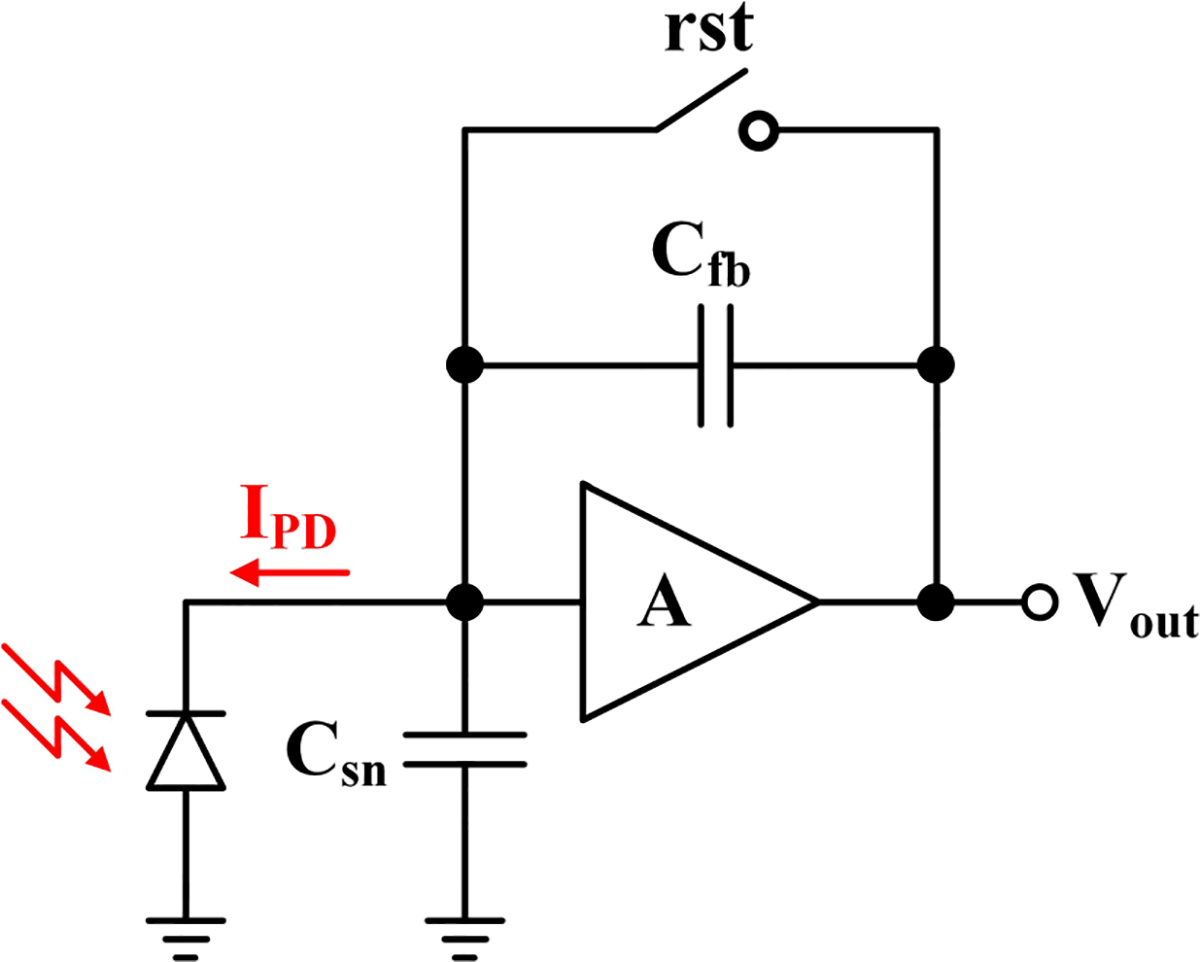
In-pixel CTIA topology.

The CTIA is a widely used circuit architecture for current-to-voltage conversion. Unlike resistive transimpedance amplifiers (TIAs), CTIAs employ a feedback capacitor instead of a resistor, allowing for charge integration over time. This design enhances the signal-to-noise ratio (SNR) and provides a better dynamic range, making CTIAs ideal for applications, such as infrared detectors, CIS, and capacitance sensing [41], [42], [43], [44]. A reset mechanism, typically implemented using an active mosfet switch, periodically discharges the feedback capacitor to prevent saturation and maintain linear operation. Due to their ability to handle low-level input currents with minimal noise, CTIAs can be used as an in-pixel amplifier [45], [46]. A CTIA consists of a high-gain inverting amplifier and a capacitance implemented in a negative feedback loop. An example of a CTIA is shown in Fig. 7.

Applying nodal analysis on Fig. 7 and rearranging the equations will express the transimpedance gain of the CTIA as presented in the following equation:

(12) $$\frac{V_{\text{out}}(s)}{I_{\text{PD}}(s)}=\frac{1}{sC_{\text{fb}}\left[1-\left(1+\frac{C_{\text{sn}}}{C_{\text{fb}}}\right)\cdot \frac{1}{A}\right]}$$

where A is the gain of the open-loop amplifier (A). Substituting with the charge instead of the current to find the CG of a CTIA-based pixel will yield the following equation:

(13) $$\text{CG}\left(\text{V}/\text{e-}\right)=\frac{q}{C_{\text{fb}}\left[1-\left(1+\frac{C_{\text{sn}}}{C_{\text{fb}}}\right)\cdot \frac{1}{A}\right]}.$$

**FIGURE 8.**
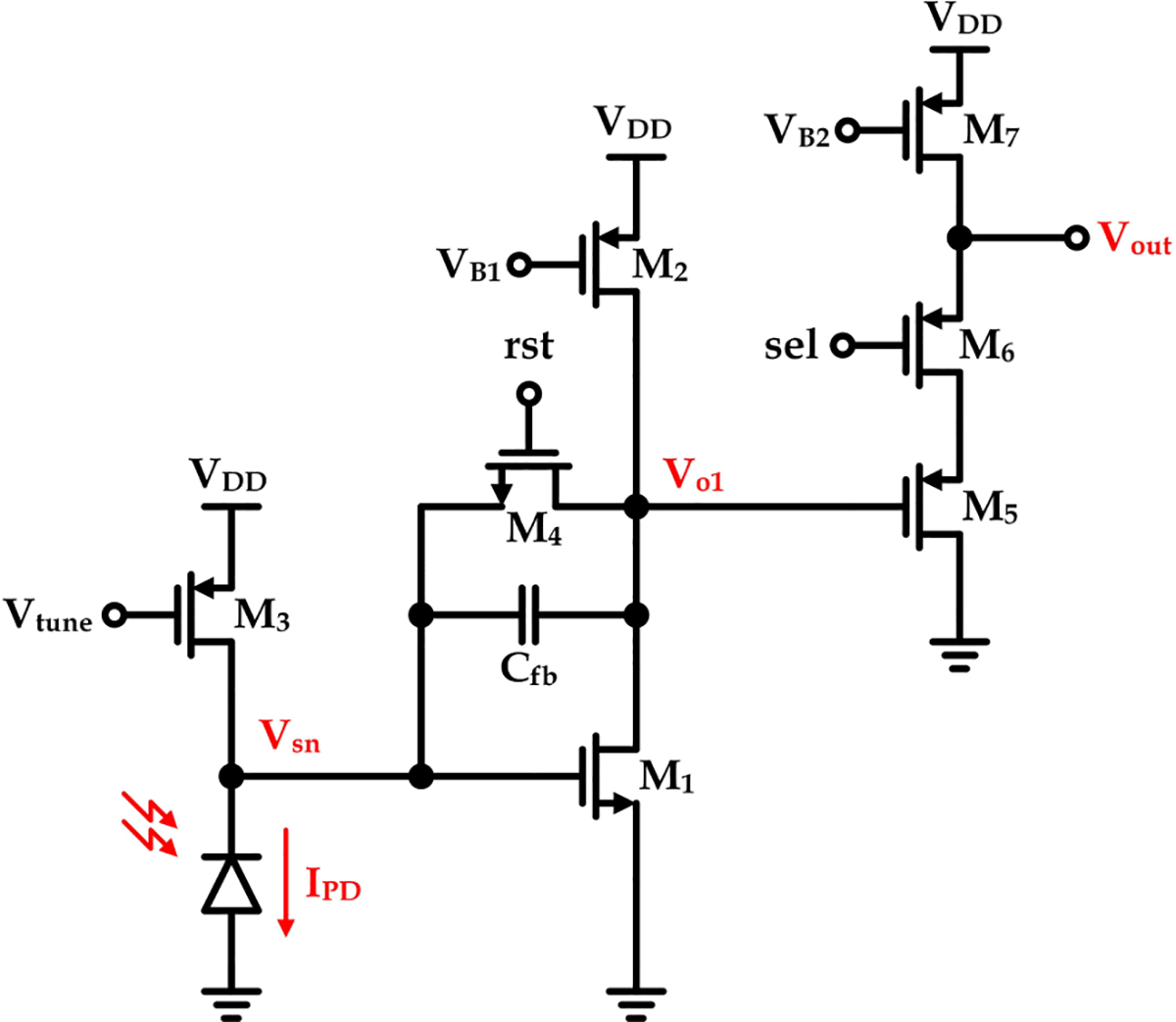
Implementation of in-pixel CTIA presented in [27].

One of the applications of in-pixel CTIA is in infrared image sensors. A dark current compensation technique was implemented for infrared detectors in [27]. In this structure, a CTIA is utilized using a single-stage open-loop CS amplifier. The gain of this circuit is determined by the ratio of the pixel's SN capacitance ($C_{\text{sn}}$) to the feedback capacitance ($C_{\text{fb}}$). Fig. 8 shows the circuit configuration used. The compensation of dark current is achieved by tuning the voltage at the gate of M3 to negate the amount of dark current before the detector is subjected to any IR radiation. After that, the net current due to the IR radiation is integrated onto $C_{\text{fb}}$. M2 acts as the active load for M1. At the end of the integration, the output signal is buffered to the column circuits using the P-type PSF amplifier formed by M5–M7. Then, $C_{\text{fb}}$ is reset or discharged by M4.

The CTIA transimpedance gain presented in (12) can be used to find the CTIA gain of the circuit in Fig. 8. The gain of the open-loop amplifier can be found in the following equation:

(14) $$A=-g_{m1}R_{\text{eq}}$$

where $R_{\text{eq}}$ is the parallel combination of the output resistances of M1 and M2. Assuming a large A, CTIA gain will depend mainly on the feedback capacitor $C_{\text{fb}}$. The CG will also depend on the feedback capacitor, which can be designed to be of smaller value than the SN capacitance. This will yield a higher CG without degrading the SN capacity. The following equations describe the transimpedance gain of the CTIA and the pixel CG assuming large A:

(15) $$\frac{V_{o_{1}}(s)}{I_{\text{PD}}(s)}\approx \frac{1}{sC_{\text{fb}}}$$

(16) $$\text{CG}(\text{V/e-})\approx \frac{q}{C_{\text{fb}}}.$$

The voltage gain expression of the in-pixel CTIA can be written as follows:

(17) $$A_{v,\text{CTIA}}=\frac{V_{o_{1}}}{V_{\text{sn}}}=-\frac{g_{m1}R_{\text{eq}}}{1+\left(g_{m1}R_{\text{eq}}\cdot \frac{C_{\text{fb}}}{C_{\text{fb}}+C_{\text{sn}}}\right)}.$$

Assuming $g_{m1}R_{\text{eq}}\gg 1$, the voltage gain of the CTIA will be reduced to be

(18) $$A_{v,\text{CTIA}}\approx -\frac{C_{\text{sn}}}{C_{\text{fb}}}.$$

The second stage of the in-pixel amplifier is a P-type SF that is used to buffer the integrated signal on $C_{\text{fb}}$ through M5. The gain of this stage is presented in (1). The CCG between the integrated photoelectrons on the SN and the pixel output voltage assuming large A is found as follows:

(19) $$A_{v,\text{SF}}=\frac{V_{\text{out}}}{V_{o1}}\approx \frac{g_{m5}\cdot R_{\text{out}}}{1+g_{m5}\cdot R_{\text{out}}}$$

(20) $$\text{CCG}(\text{V}/\text{e}-)=\frac{V_{\text{out}}}{Q_{\text{in}}}\approx \frac{q}{C_{\text{fb}}}\cdot \left(\frac{g_{m5}\cdot R_{\text{out}}}{1+g_{m5}\cdot R_{\text{out}}}\right)$$

where $R_{\text{out}}$ is the parallel combination of M5 and M7 resistors, assuming on resistance of M6 is negligible. Reduction of the feedback capacitance $C_{\text{fb}}$ will lead to increased gain. However, the reset noise will limit its minimum value. The PSF's gain depends on the output impedance of the pixel and hence will reduce the effective gain. The overall voltage gain of such a circuit can be concluded from (18) and (19) in the following equation:

(21) $$A_{v}=\frac{V_{\text{out}}}{V_{\text{sn}}}\approx -\frac{C_{\text{PD}}}{C_{\text{fb}}}\cdot \left(\frac{g_{m5}\cdot R_{\text{out}}}{1+g_{m5}\cdot R_{\text{out}}}\right).$$

The design in [46] requires the use of an external voltage to try to cancel the dark current, which complicates the design and makes it subject to external noise. An array of 32 × 29 pixels was fabricated in a 0.8-μm CMOS process technology with a $55\times 50\;\mu {\text{m}}^{2}$ pixel size. The pixel gain was reported to be 42dB≈125V/V, and the fixed pattern noise (FPN) was $2.6\;{\text{mV}}_{\text{rms}}$. The type of detector used was an N-well, P-substrate photodiode.

**FIGURE 9.**
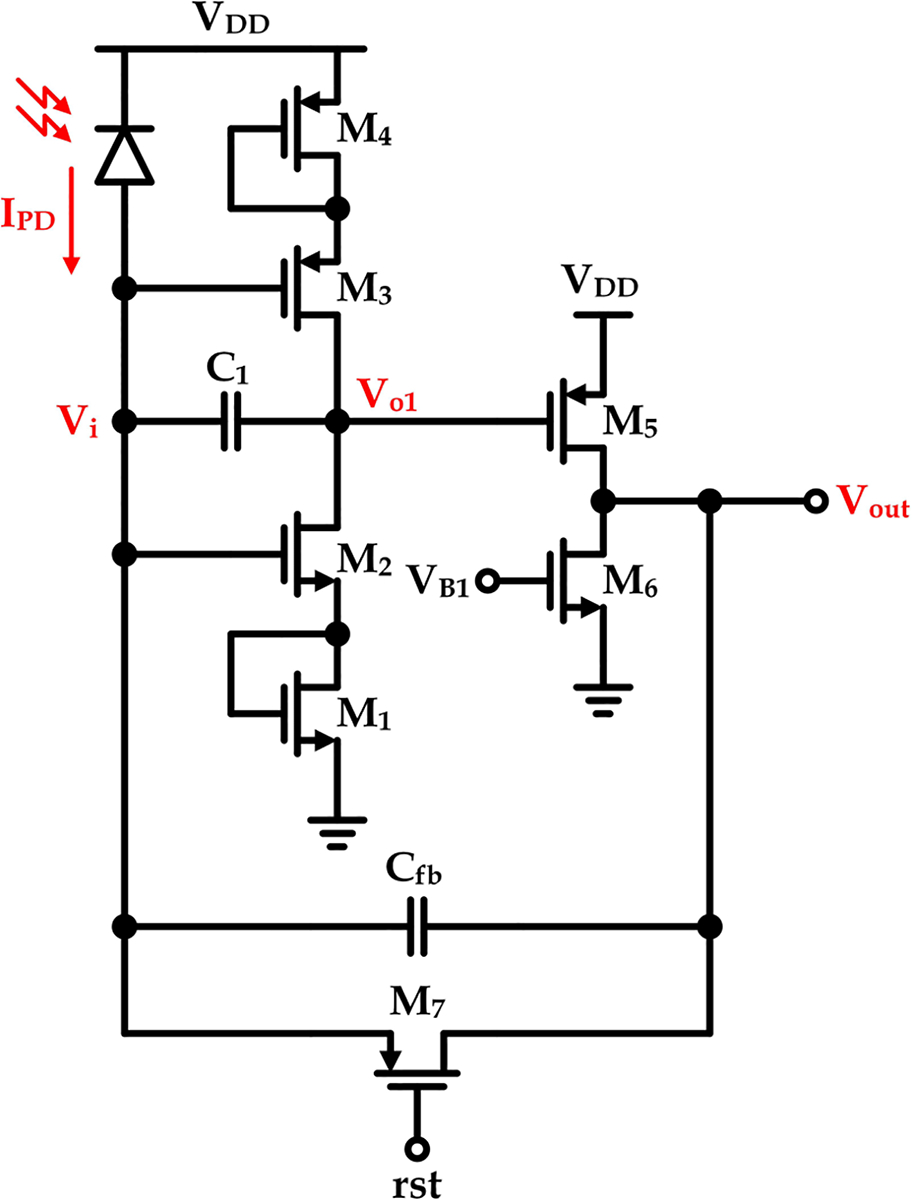
In-pixel closed loop push–pull amplifier proposed in [47] for X-ray imaging.

In 2001, Abdalla et al. [47] proposed a closed-loop in-pixel CTIA amplifier for X-ray imaging applications. X-ray imaging is widely used in medical imaging, including bone fracture detection and dental imaging [48]. The CTIA pixel design incorporates a push–pull CS preamplifier with an integration feedback capacitor followed by an SF. The corresponding pixel circuit is illustrated in Fig. 9. Utilizing a P-/N-well photodiode under scintillator coating offers advantages for X-ray detection, as its low quantum efficiency results in reduced absorption of direct X-rays [47]. In addition, since the photodiode's anode is not directly connected to the substrate, it minimizes noise pickup compared to conventional photodiode structures.

Transistors M1–M4 constitute the push–pull TIA. After amplification, M5 buffers the signal to the column circuits. The amplifier's output is fed back to the input via the feedback capacitor $C_{\text{fb}}$. During the integration phase, the input current is accumulated on $C_{\text{fb}}$, functioning similarly to a CTIA amplifier. Once the integration and readout phases are complete, M7 resets the feedback capacitor, initiating the next integration cycle.

A 32 × 80 pixel array was fabricated using a 0.8-μm CMOS double-poly, n-well process with a pixel pitch of 50μm. The proposed pixel amplifier achieved a gain of 30 V/V and an SNR of 52.3 dB. The overall gain of this architecture can be determined as follows:

(22) $$A_{V}\approx -\frac{C_{\text{sn}}}{C_{\text{fb}}}.$$

To meet HDTV cameras' low random noise requirements, Kozlowski et al. [49], [50] introduced a novel technique to reduce the reset noise in three transistor (3T) APS. In this approach, the 3T-APS operates like a CTIA during the reset phase and transitions to an SF amplifier during readout. Instead of functioning as an ideal switch, the reset switch acts as a variable resistor through a tapered reset scheme. The design leverages the parasitic gate-to-drain capacitance of the CS stage as the feedback capacitance of the CTIA, achieving a dc voltage gain of 18 dB during the reset phase. By optimizing the reset switch resistance, feedback capacitance, and amplifier output resistance, reset noise is minimized without requiring CDS. A 1920 × 1080 pixel array was fabricated using a 0.25-μm CMOS process, with a reported pixel pitch of 5μm. The pixel reset noise was reduced to 3 e–, while the pixel CG was measured to be 32μV/e-.

**FIGURE 10.**
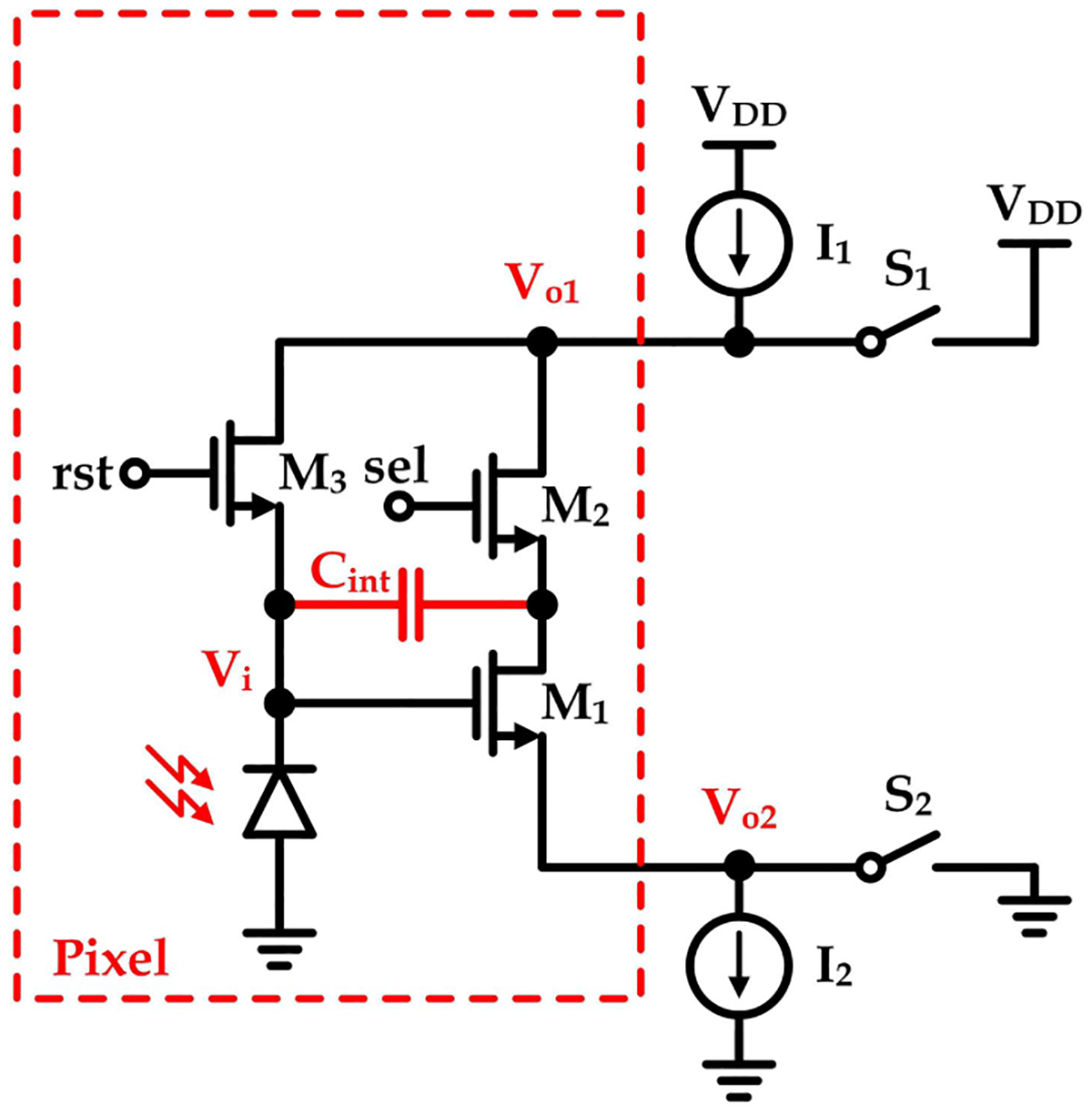
3T-APS as proposed by Yang et al. [45].

In 2006, Yang et al. [45] proposed a 3T-APS pixel with a configurable in-pixel CTIA-SF architecture. The corresponding pixel circuit is illustrated in Fig. 10. This design allows the 3T pixel transistors to operate in either a CS or CD configuration, depending on image brightness. The column circuits include two bias currents and two switches to support both configurations.

In the CS mode, the operation begins by resetting a row of pixels while enabling the select switch. Afterward, both switches are turned off, allowing the photogenerated charge to integrate on the photodiode capacitance. At the end of the integration phase, the select transistor is switched on again, and amplification occurs as the integrated electrons are transferred to the feedback capacitor. Assuming a very high open-loop voltage gain, the CS amplifier behaves as a CTIA, where the feedback capacitance is the gate-to-drain capacitance of M1. In addition, the select transistor can function as a cascode transistor to further reduce the feedback capacitance and mitigate the Miller effect.

The photoresponse voltage depends on $C_{gd2}$, which is significantly made smaller than the SN capacitance. As a result, high responsivity can be achieved for low-light images. Conversely, in high-brightness scenes, the high gain of a CTIA is unnecessary and could lead to image saturation. To prevent this, the three transistors are reconfigured to operate as an SF amplifier when bright images are detected.

A 2140 × 1584 pixel array was fabricated using a 0.18-μm CMOS CIS technology node, with a pixel pitch of 2.54μm. The pixel achieved a CCG of $110\;\mu \text{V}/{\text{e}}^{-}$ and a read noise of $3{\text{e}}^{-}$.

CTIA was used in many other research endeavors to enhance low-light imaging of the image sensors. One of which is the fluorescence imaging that has emerged as a robust method for carrying out minimally invasive spatiotemporal mapping of the structure and function of cellular and neural systems [51].

**FIGURE 11.**
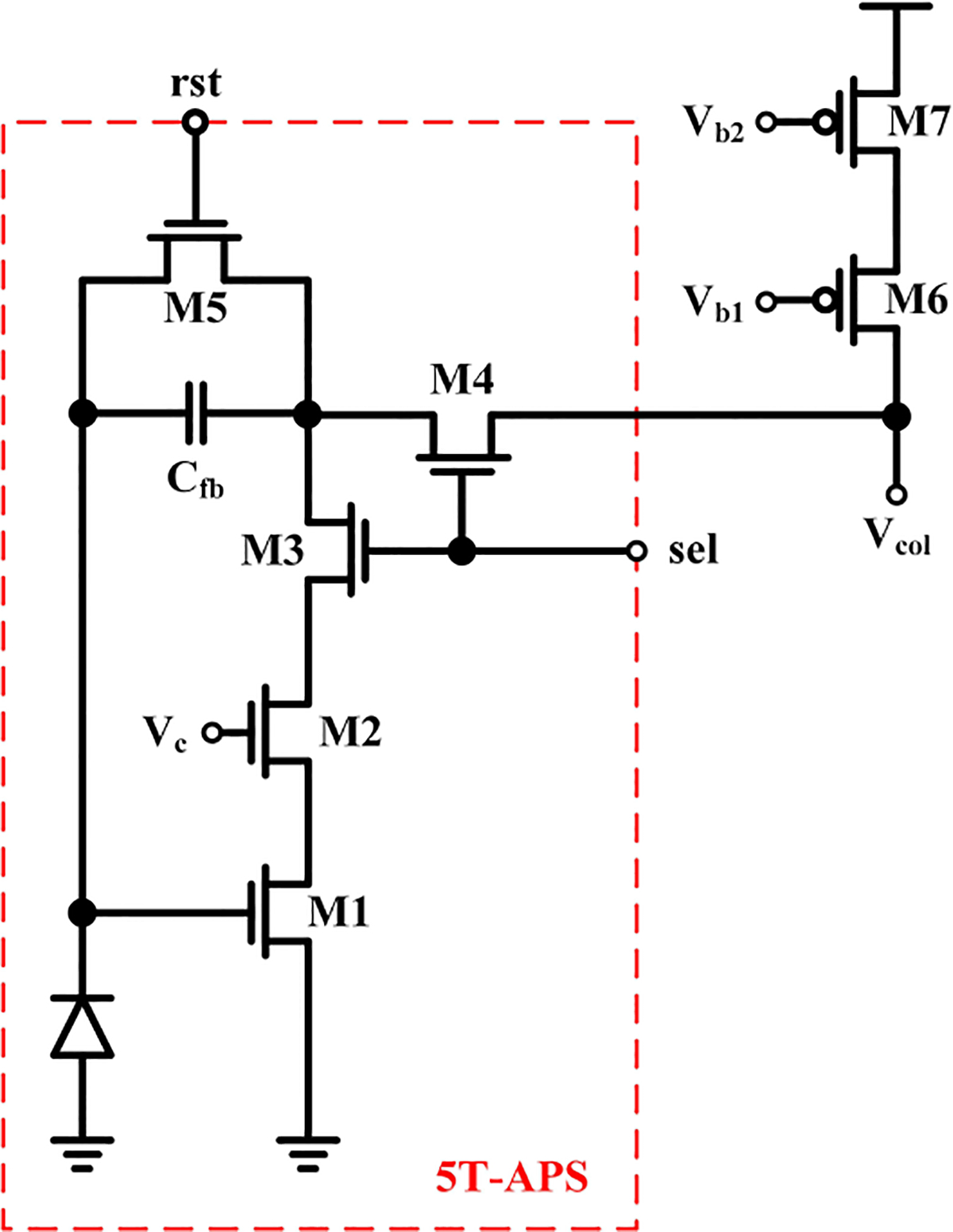
5T CTIA proposed by Murari et al. [46].

In 2011, Murari et al. [46] employed a CTIA pixel to improve the sensitivity of a fluorescence imager. The pixel design incorporated five negative channel metal oxide semiconductor fiel effect transistor (NMOS) transistors (5T), a feedback capacitor, and a photodiode, as illustrated in Fig. 11. In addition, a delta differencing circuit was utilized to compute the difference between the reset signal and the photogenerated signal [52]. Due to the inclusion of five transistors, a feedback capacitor, and the need for a high open-loop voltage gain, the pixel area was expanded to approximately $400\;\mu {\text{m}}^{2}$.

The circuit is implemented using a 0.5-μm standard CMOS technology. A pixel array of 132 × 124 with a pixel pitch of 20.1μm was fabricated. The CTIA's voltage gain was reported as 50 V/V, achieved by using a small feedback-integration capacitor relative to the SN capacitance, with values of $C_{\text{fb}}=5\;\text{fF}$ and $C_{\text{sn}}=250\;\text{fF}$. The CCG was $32\;\mu \text{V}/{\text{e}}^{-}$ based on the reported design parameters. In addition, the input-referred read noise was measured at $26\;{\text{e}}^{-}$, while the reset noise was $6\;{\text{e}}^{-}$.

### E. IN-PIXEL LOGARITHMIC AMPLIFIER

**FIGURE 12.**
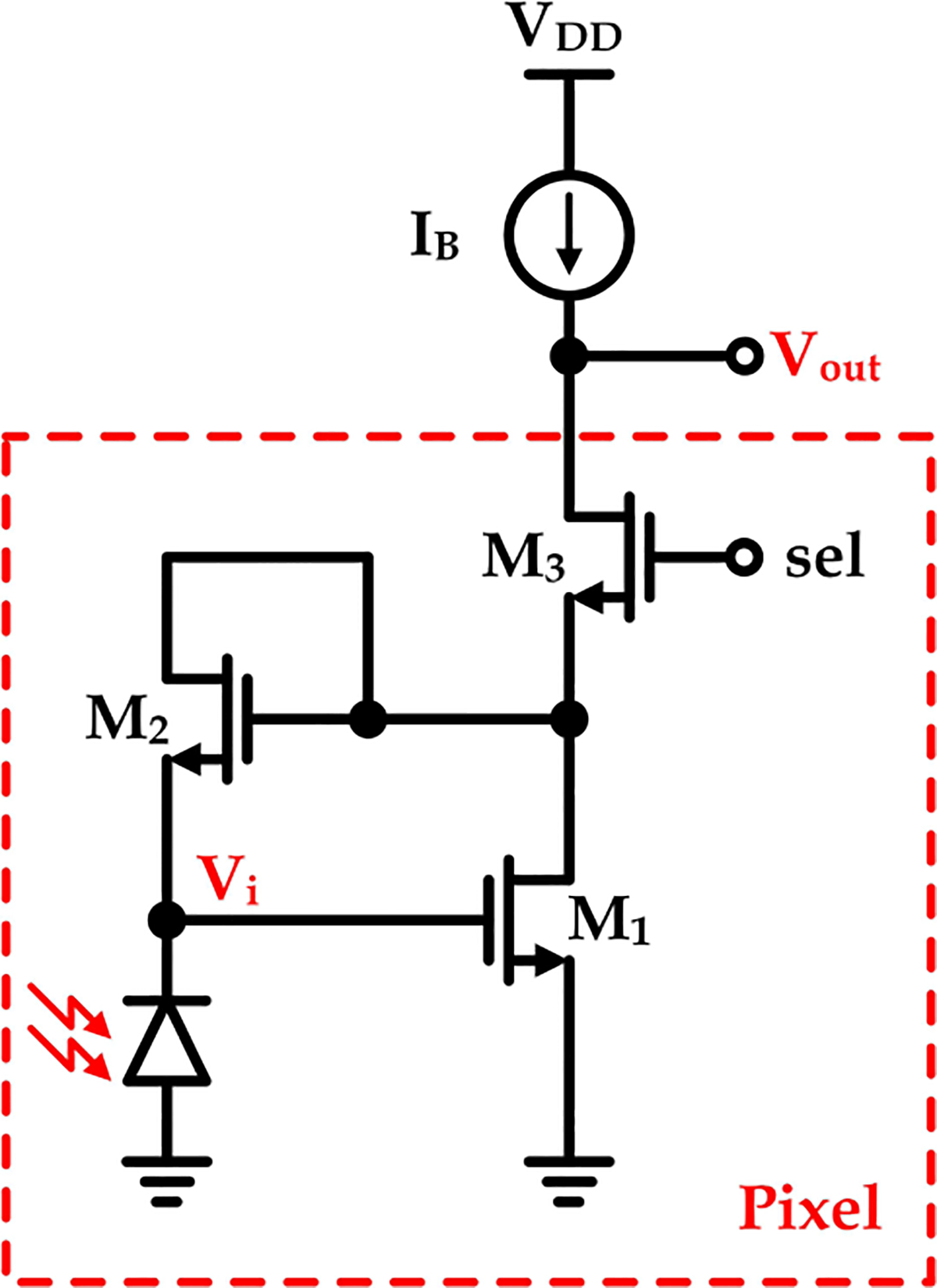
In-pixel log-amp operating in weak inversion mode [53].

Hauschild et al. [53] proposed using a logarithmic in-pixel amplifier (log-amp) working in the subthreshold region, as seen in Fig. 12. Hauschild was working on implementing a retina implant system for partially sighted patients suffering from retinal pigmentosa [54]. In implementing such a system, an image acquisition method is required. A pixel array is used for this purpose. It is required to have a large dynamic range (high-brightness image capture) for the input to match the dynamic range of the retina. For that reason, a log-amp was used in the pixel structure. Benefiting from the logarithmic relation between drain current and voltage of a mosfet in the weak inversion mode, an amplifier can sense very bright images and generate small voltages increasing the dynamic range of the pixel. Within the context of this circuit, M1 serves as the primary transistor of the CS amplifier and is a native device possessing a threshold voltage (V th) of −0.2 V. M2 transistor serves as a feedback element, connecting the output of the amplifier to its input. M3 serves as the column switch, facilitating the transmission of signals from the amplifier to the sensor's output. This design capitalizes on the logarithmic correlation between current and voltage exhibited by MOS transistors operating in the subthreshold (weak inversion) regime, as with the following equation:

(23) $$V_{o}=V_{To}+nV_{S}+10nV_{t}\cdot \text{ln}\left(\text{log}\frac{I_{D}}{I_{S}}\right)$$

where $V_{G}$ and $V_{S}$ are the gate and source voltages of the transistor M1, respectively, $V_{t}$ is the thermal voltage, and $I_{S}$ in the order of 100 nA.

The pixel dimensions of this design were 38.8μm×33.6μm in a pixel array of 32 × 32 pixels fabricated using a 1.5-μm CMOS process. The area of the pixel array was 7 mm^2^. The gain of the amplifier and its CG was not reported. However, the ratio between the photodiode's output voltage and input current is 100 mV/decade, achieving the pixel's wide dynamic range. Schanz et al. [55] stated the output noise voltage of the proposed structure to be as follows:

(24) $${\overset{‾}{v}}_{\text{out},n}^{2}=\frac{2}{3}\cdot \frac{\left(1+n+\frac{g_{m2}}{g_{m1}}\left(1+\frac{g_{m3}}{g_{m1}}\right)\right)}{\left(C_{\text{sum}}+C_{\text{sensor}}\cdot \frac{g_{m3}}{g_{m1}}\right)}$$

in which $C_{\text{sum}}$ is the parallel combination of the gate-to-source capacitance of M2 and the gate-to-drain capacitance of M1. $C_{\text{sensor}}$ is the photodiode capacitance and the gate-to-source capacitance of M1, and n is the subthreshold factor.

### F. DIFFERENTIAL INPUT IN-PIXEL AMPLIFIERS

**FIGURE 13.**
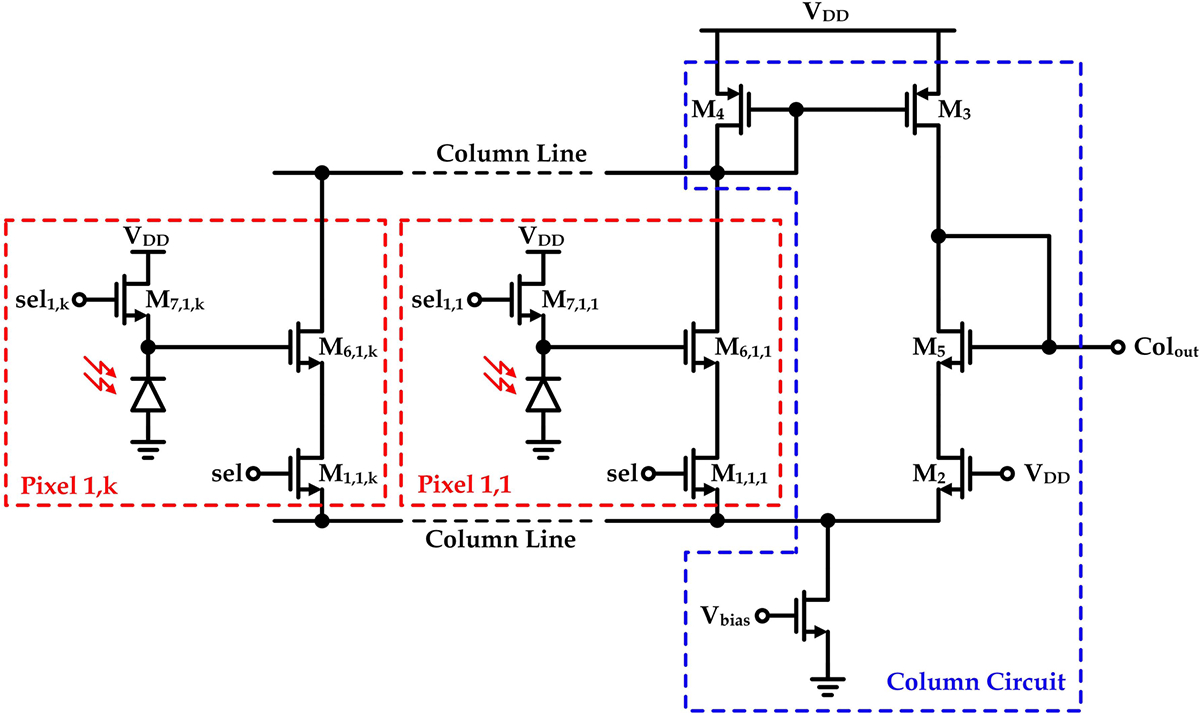
Active column sensing technique as presented in [56].

Up to this point, the pixel design revolved around single-stage amplifier configurations, mainly the open-loop SF amplifier or closed-loop CS amplifiers. In 2000, Vogelsong et al. [56] developed a new technique to use a true unity-gain amplifier rather than the nonlinear SFs to buffer the pixel signals. The technology called active column sensing (ACS) employed a differential amplifier in a unity-gain configuration, as shown in Fig. 13.

The active column sensing (ACS) technique divides the differential amplifier into two parts: the pixel will contain only two transistors of the differential amplifier plus the reset switch, while the rest of the differential amplifier will be common to all pixels of a single column. By using this technique, the fill factor and area requirement will be the same as in standard 3T-APS while enhancing the image quality and reducing the FPN associated with PSF image sensors. Using the differential amplifier improves the FPN performance of the pixel, and the overall sensor's noise is reduced.

The ACS was implemented in a 0.5-μm CMOS technology. A pixel array of 1000 × 1000 pixel was implemented with a pixel pitch of 12μm. This configuration can achieve a CG of 10μV/e-.

**FIGURE 14.**
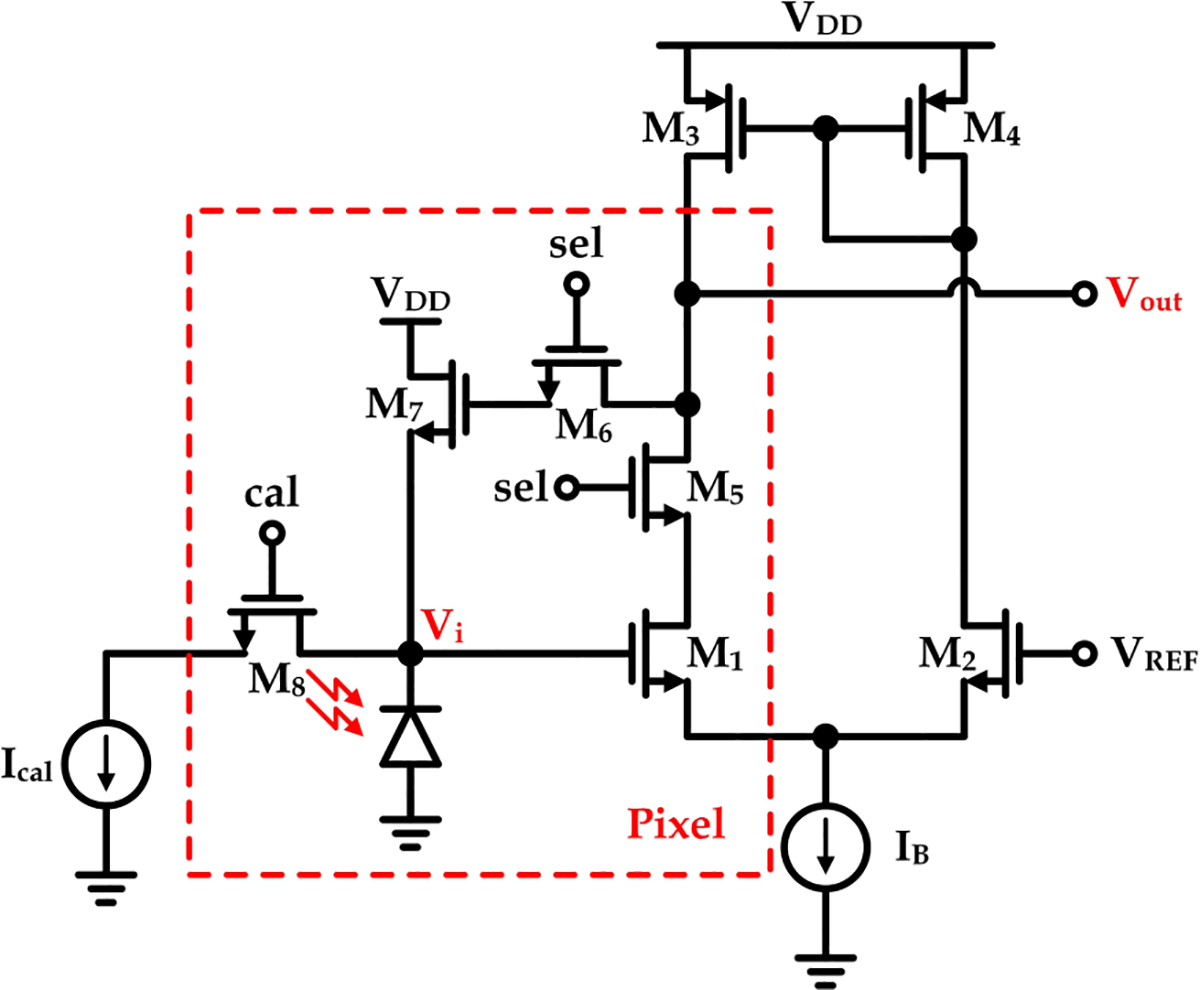
Partly in-pixel differential amplifier with feedback as proposed in [57].

Two years later, Graupner et al. [57] used ACS to implement a CIS with dark current calibration technique. The dark current and the FPN are suppressed in this design by adding an external calibration current source, as shown in Fig. 14. Instead of using unity-gain feedback as in [56], Graupner et al.[57] suggested biasing the differential amplifier in weak inversion mode. Benefiting from the logarithmic relationship between gate voltage and drain current of mosfet biased in weak inversion. This circuit addresses the dark current variation problem with the varying reverse bias voltage. The idea of the feedback is to stabilize the reverse voltage over the photodiode to avoid increasing dark current with the increase in reverse voltage. If the gate voltage of M2 decreases, its drain voltage will increase, and the gate-to-source voltage of M1 will increase, increasing the drain current, which in turn increases the voltage over the photodiode as a counteraction to the decrease in M2 gate voltage. The proposed amplifier exhibits a logarithmic relationship between the current and voltage; hence, a wide dynamic range was achieved. The following equation describes the relationship between the output voltage and integrated current [57]:

(25) $$V_{\text{out}}=V_{\text{REF}}+V_{t1}+nV_{T}\;\text{ln}\left(\frac{I_{\text{ph}}+I_{\text{dark}}}{I_{B}}\right).$$

Here, $I_{\text{ph}}$ represents the integrated current over the photodiode cathode, while $I_{\text{dark}}$ denotes the dark current associated with the pixel. $V_{t1}$ is the threshold voltage of transistor M1, n is the subthreshold region factor, and $V_{T}$ is the thermal voltage. It is worth mentioning that both Hasuchild et al. [53] and Graupner et al. [57] employed the same approach, utilizing either a single-stage amplifier or a differential amplifier. Despite the partitioning of the differential pair, the pixel's fill factor remains low, reported to be 27%. The fabricated pixel array had dimensions of 288(H) × 352(V) with a pixel pitch of 15μm. However, neither the CG nor the read noise was reported.

Another research involving ACS was reported in [36]. This work compares four types of amplifier implementations using the ACS technique. They employed soft and hard reset, and P- and N-type differential pairs. All the designs were based on implementing a unity-gain operational amplifier similar to that proposed by Vogelsong et al. [56]. The goal of this work was to improve the characteristics of the OPAMP. It was reported that using P-channel OPAMP will have a better slew rate, higher unity-gain frequency, and an excellent detection of high illumination (low voltage) and accepted detection of low illumination (high voltage) compared to N-channel OPAMP, which is better in terms of the fill factor. Using hard or soft reset would reduce the image lag (or increase it) and will determine the output swing. However, the closed-loop gain of the in-pixel amplifier is still around unity.

Each of the four designed amplifier types was implemented using a 0.35-μm CMOS technology, with each type featuring a pixel array of 16 × 64. The CCG ranged from a minimum of $6.5\;\mu \text{V}/{\text{e}}^{-}$ (soft reset, P-channel) to a maximum of $11.4\;\mu \text{V}/{\text{e}}^{-}$ (hard reset, N-channel). The pixel pitch was 7μm across all four types. However, no read noise was reported for any of them.

**FIGURE 15.**
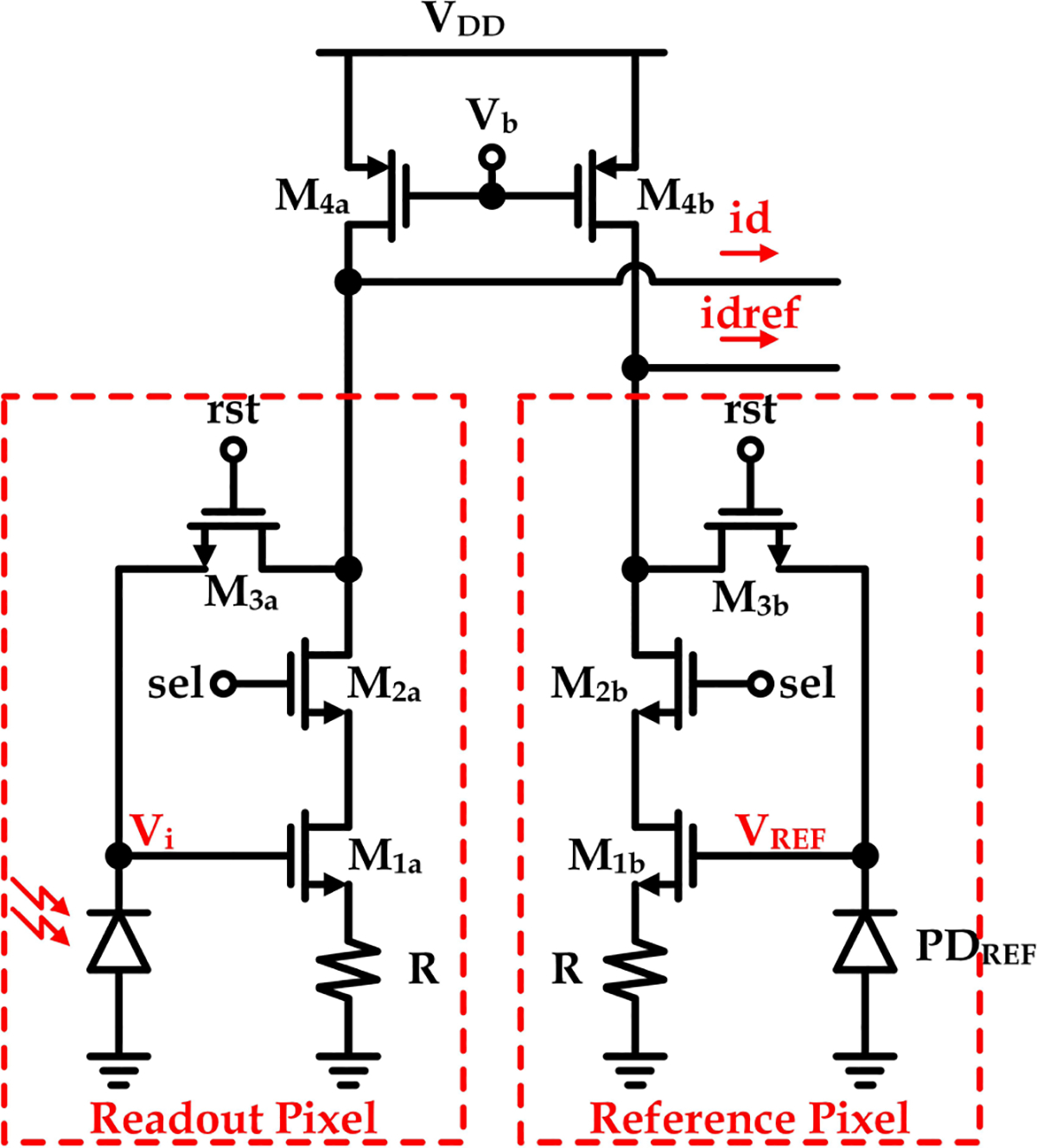
Pseudodifferential photodiode pixel as reported by Huang et al. [59].

Fluorescence spectroscopy is one of the widely used techniques in biomedical research. A CIS was designed for fluorescence imaging applications in [58] and [59]. The pixel structure for this sensor was a pseudodifferential one to improve the noise performance of the pixel. The structure is shown in Fig. 15. The voltages on the gate of M1a and M1b are converted to current and fed differentially to a column-level TIA, which converts and amplifies the difference. The architecture described in this study utilizes the active reset technique, which involves the implementation of a high-gain amplifier in a negative feedback configuration. This configuration allows for precise sensing and comparison of variations in the voltage of the reset photodiode against a reference waveform. Simultaneously, it effectively suppresses these fluctuations by regulating the opposing reset current of the reset transistor responsible for charging. This technique was originally proposed by Pain et al. [60] and has been widely adopted due to its ability to significantly enhance the noise performance of the pixels with the addition of just one transistor [8], [60], [61], [62], [63], [64].

A 64 × 64 pixel array was designed using this pixel structure, featuring a pixel pitch of 50μm in a 0.18-μm CMOS process. The input-referred read noise was $12.9\;{\text{e}}^{-}$, while the reset noise measured was $186.4\;{\text{e}}^{-}$. The CCG was $8\;\mu \text{V}/{\text{e}}^{-}$.

**FIGURE 16.**
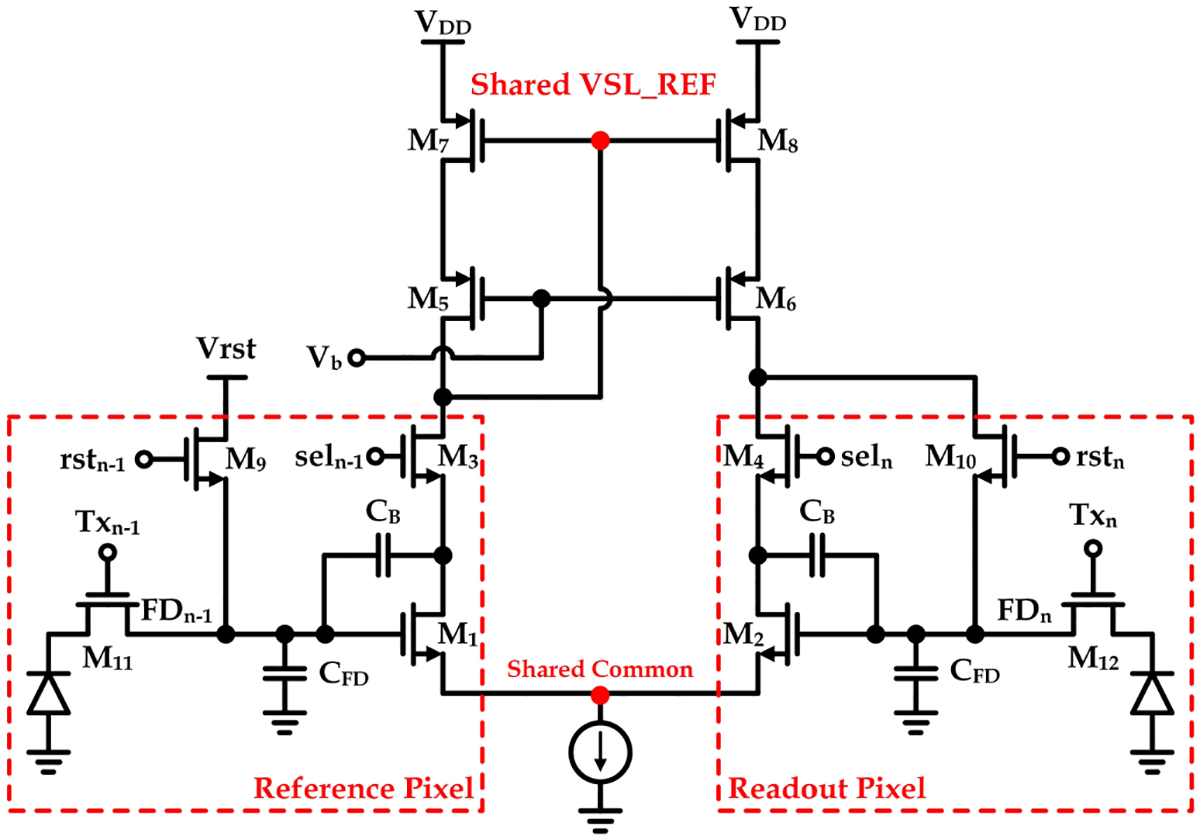
Differential in-pixel amplification using reference pixel as well as the readout in a 3-D stacked image sensor [8].

A low-noise, high dynamic range, and small pixel area CIS was proposed by Sato et al. [8], where a reference shared in-pixel differential amplifier was adopted, as shown in Fig. 16. In this design, each adjacent two rows of pixels form a pixel to be measured and another to be the reference. So, if the first row is being read out, the second row will act as a reference to the first row, and so on. This technique forms a differential amplifier using two pixels, reducing the pixel's area. The tail current source of the differential amplifier is connected in common to all the columns as well as the cascoded wideswing PMOS current mirror load. This pixel can be configured to be used either as an SF or a CS in a differential fashion with switch controls on the column side of the readout without adding extra transistors in the pixel itself. The following equation can model the CCG for such a pixel [8]:

(26) $$\text{CCG}(\text{V}/\text{e}-)=\frac{q}{\left(\frac{C_{\text{sn}}+C_{B}}{A_{v}+C_{B}}\right)}$$

where $A_{v}$ is the low-frequency open-loop voltage gain of the in-pixel amplifier, $C_{B}$ is the feedback capacitor, $C_{\text{sn}}$ is the floating diffusion capacitance, and q is the electron charge. In this design, $C_{B}$ is chosen to be much smaller than $C_{\text{sn}}$; hence, the CCG can be maximized and approximated by the following equation:

(27) $$\text{CCG}(\text{V}/\text{e}-)\approx A_{v}\cdot \frac{q}{C_{\text{FD}}}.$$

The differential gain of this structure is approximately 10 V/V with a CG of 75μV/e- for SF configuration and a CCG of 560μV/e- for the CS configuration. The pixel array size is 3840(H) × 2160(V) with a pixel pitch of 1.45μm using a 90-nm CMOS BSI-CIS process.

The reduced pixel pitch of the sensor is due to using a stacked technology in which two dies can be bonded together with Cu–Cu connections. The extra wiring does not affect the sensor's sensitivity since a back-side illumination (BSI) technology is used for fabrication.

### G. OTHER IN-PIXEL AMPLIFIERS

**FIGURE 17.**
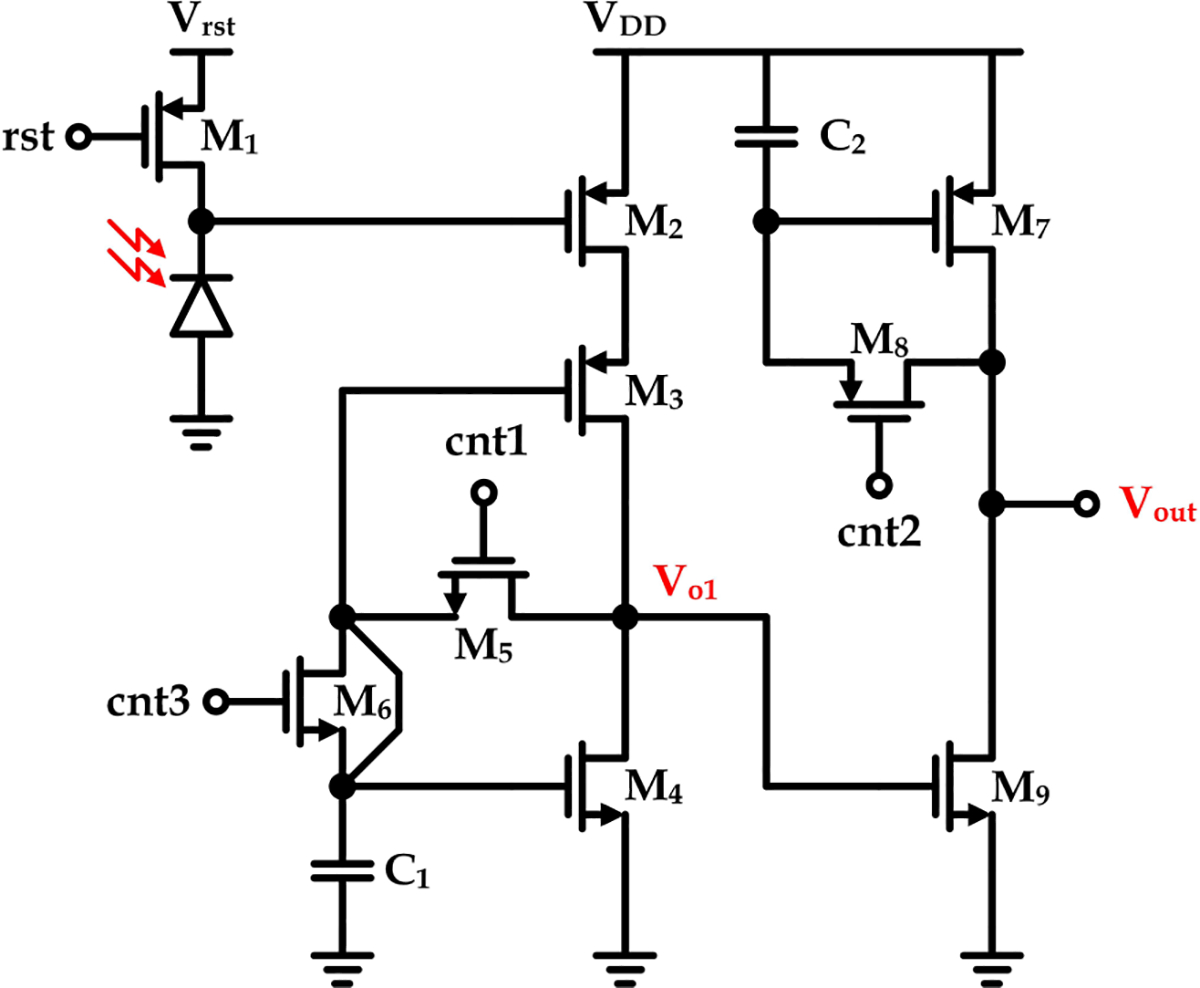
Two-stage in-pixel amplification proposed by Pain and Fossum [65].

In 1996, Pain and Fossum [65] proposed and demonstrated two self-biased in-pixel amplifier circuits. The first one is illustrated in Fig. 17. The circuit configuration has two amplification stages: a P-type cascode CS amplifier and an N-type CS amplifier, adopting a self-biasing scheme. The circuit's operation is divided into two distinct phases. During phase 1, the photodiode is reset to the reference voltage ($V_{\text{rst}}$) by activating M1. In phase 2, the activation of M5 enables the connection between the gates and drains of M3 and M4. This connection is crucial for achieving the maximum gain of the pixel amplifier. The bias voltage is stored on capacitor $C_{1}$, which effectively maintains the voltage at the gates of M3 and M4, corresponding to the highest achievable gain. Meanwhile, M8 employs a conceptual framework similar to M5, which influences M7 appropriately. A dummy switch is used for compensation to avoid the charge injection error from M5. The gain equations for the self-cascoded amplifier are reported to be [66]

(28) $$A_{v_{1}}\approx -\frac{g_{m2}\cdot g_{m3}}{g_{ds4}}(1+\eta )$$

(29) $$A_{v_{2}}=\frac{g_{m9}}{g_{ds9}+g_{ds7}}$$

(30) $$A_{v_{t}}=-\frac{g_{m2}\cdot g_{m3}\cdot g_{m9}}{g_{ds4}\left(g_{ds9}+g_{ds7}\right)}(1+\eta )$$

where η is the ratio between the body transconductance and the gate transconductance of transistor M3, $A_{v_{1}}$ and $A_{v_{2}}$ are the voltage gain of the first and second stages, respectively. The reported gain of the pixel using the two-stage in-pixel amplifiers was around 63 dB, equivalent to a voltage gain of 1400 V/V. Utilizing a larger capacitance ($C_{1}$) in the amplifier circuitry is more effective in reducing the input-referred noise, as opposed to relying on the smaller capacitance of the photodiode SN. Nevertheless, a tradeoff exists among area, speed, and noise factors. The output-referred dominant noise source of the first stage amplifier is approximately reported as follows for this design [65]:

(31) $${\overset{‾}{v}}_{\text{rst}}^{2}=\frac{KT}{q}\left(1+\frac{1}{g_{m4}\cdot r_{ds5}}\right).$$

**FIGURE 18.**
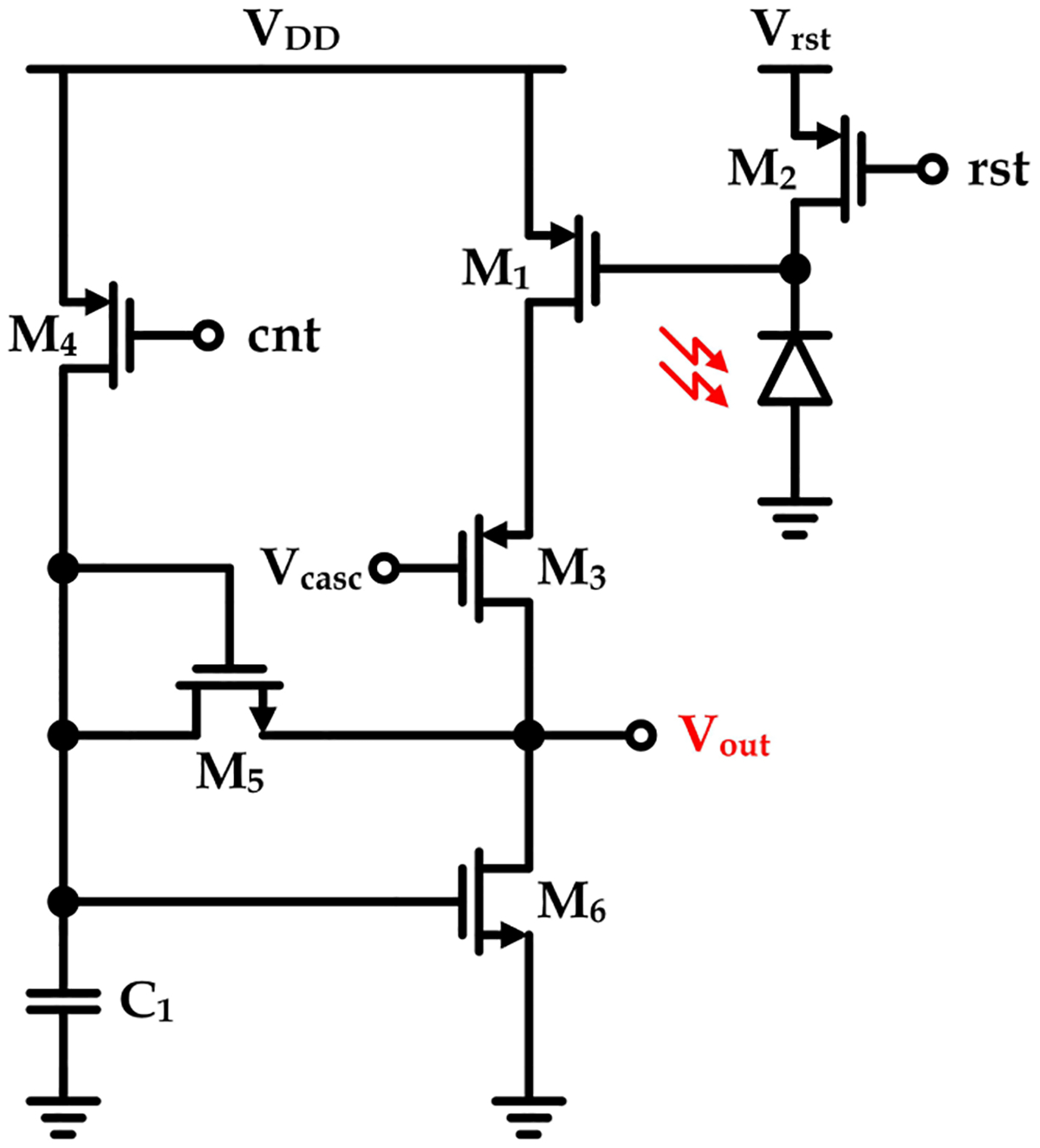
Two-stage in-pixel amplification proposed by Pain and Fossum [65].

Fig. 18 shows the second pixel suggested in [65]. In this design, the complexity is slightly diminished by employing a single-stage amplifier rather than two, thereby reducing the occupied area. This design employs a singular clock signal to provide the most optimal biasing of the amplifier. Similar to the first circuit, the operation of the circuit is partitioned into two distinct phases: a reset phase and an amplification phase. During the amplification phase, transistor M4 activates, while the gate of transistor M5 is biased with a voltage equal to $V_{DD}$ minus the threshold voltage of the n-type MOS transistor. This condition results in a significantly reduced $V_{\text{out}}$. When the M4 transistor is deactivated, a current is established from the drain to the source of the M5 transistor, resulting in the discharge of the voltage on capacitor $C_{1}$. This discharge causes an increase in the output voltage, $V_{\text{out}}$, until the M5 transistor is also deactivated. Following the conclusion of this phase, it is expected that M6 will exhibit a bias toward its high-gain optimal point. The reported gain of this amplifying circuit was approximately 34.8 dB, equivalent to a voltage gain of approximately 60 V/V. The gain of the amplifier is divided into two steps. The first step is the gain from the input of the amplifier to the output of M1, while the second step takes the output of M1 and amplifies it with the cascode device M3. The following equations describe the gain for the amplifier [66]:

(32) $$A_{v_{1}}=-\frac{2\cdot g_{m1}}{g_{m3}\cdot (1+\eta )}$$

(33) $$A_{v_{2}}=\frac{g_{m3}}{g_{ds3}}(1+\eta )$$

(34) $$A_{v_{t}}\approx -\frac{g_{m1}\cdot g_{m3}}{g_{m6}}(1+\eta )$$

where η is the ratio between the body transconductance and the gate transconductance of transistor M3. To avoid high miller capacitance associated with transistor M1, $A_{v1}$ should be minimized, while $A_{v2}$ should be maximized to achieve the required high gain [67].

**FIGURE 19.**
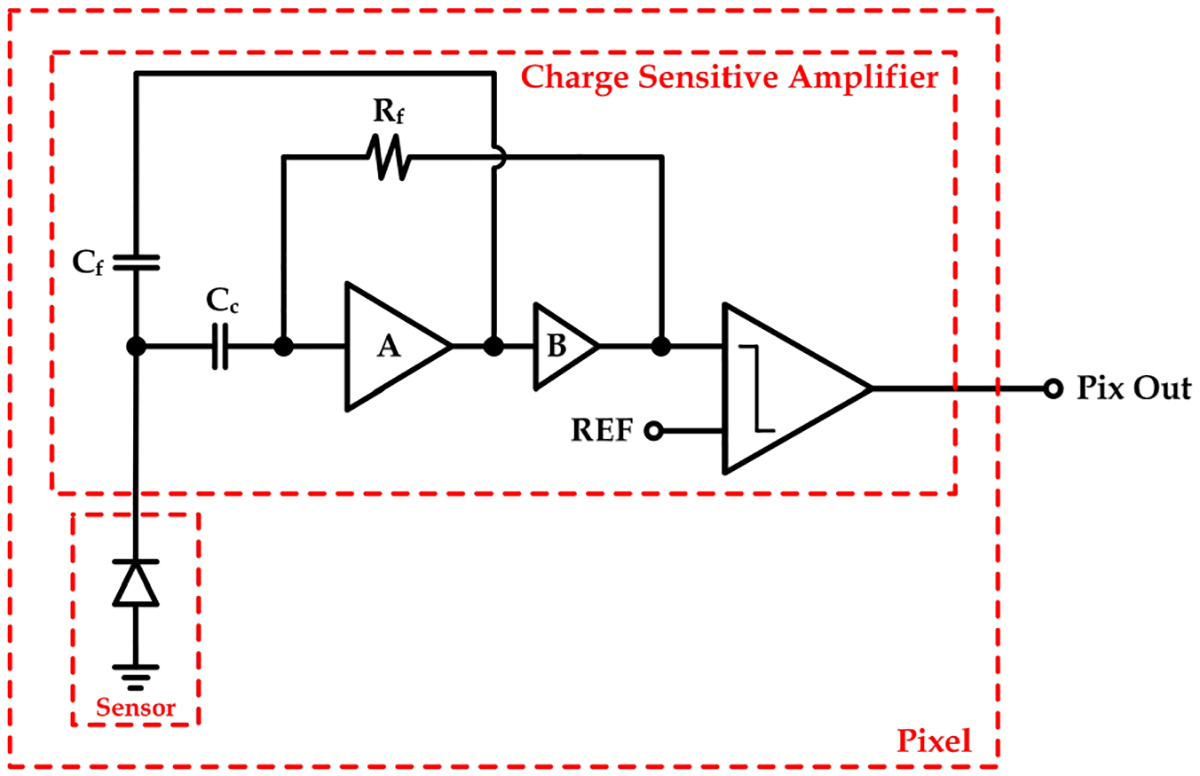
High-voltage APS block diagram in [26].

High-voltage CIS is widely used today in experimental particle physics, electron microscopy, and particle therapy beam monitoring applications. High-voltage CIS can be implemented by using deep N-wells that help isolate all the readout electronics in a low-doped isolated P-substrate. This structure allows biasing of the substrate with a high negative voltage (50–150 V) without affecting the readout electronics and results in a wide depletion region. In 2021, Peric et al. proposed [26] a high-voltage APS where the substrate was biased at −50 V and the depletion region width was 30μm. This design used a folded cascode operation amplifier as a charge sense amplifier in a negative feedback configuration as in a CTIA. The theoretical gain of this amplifier is about 150 V/V. The block diagram of the pixel is shown in Fig. 19. The pixel area is 150μm×50μm, and an array of 132(H) by 372(V) pixels is fabricated in a 180-nm HVCMOS process.

## III. PERFORMANCE EVALUATION AND COMPARISON

**FIGURE 20.**
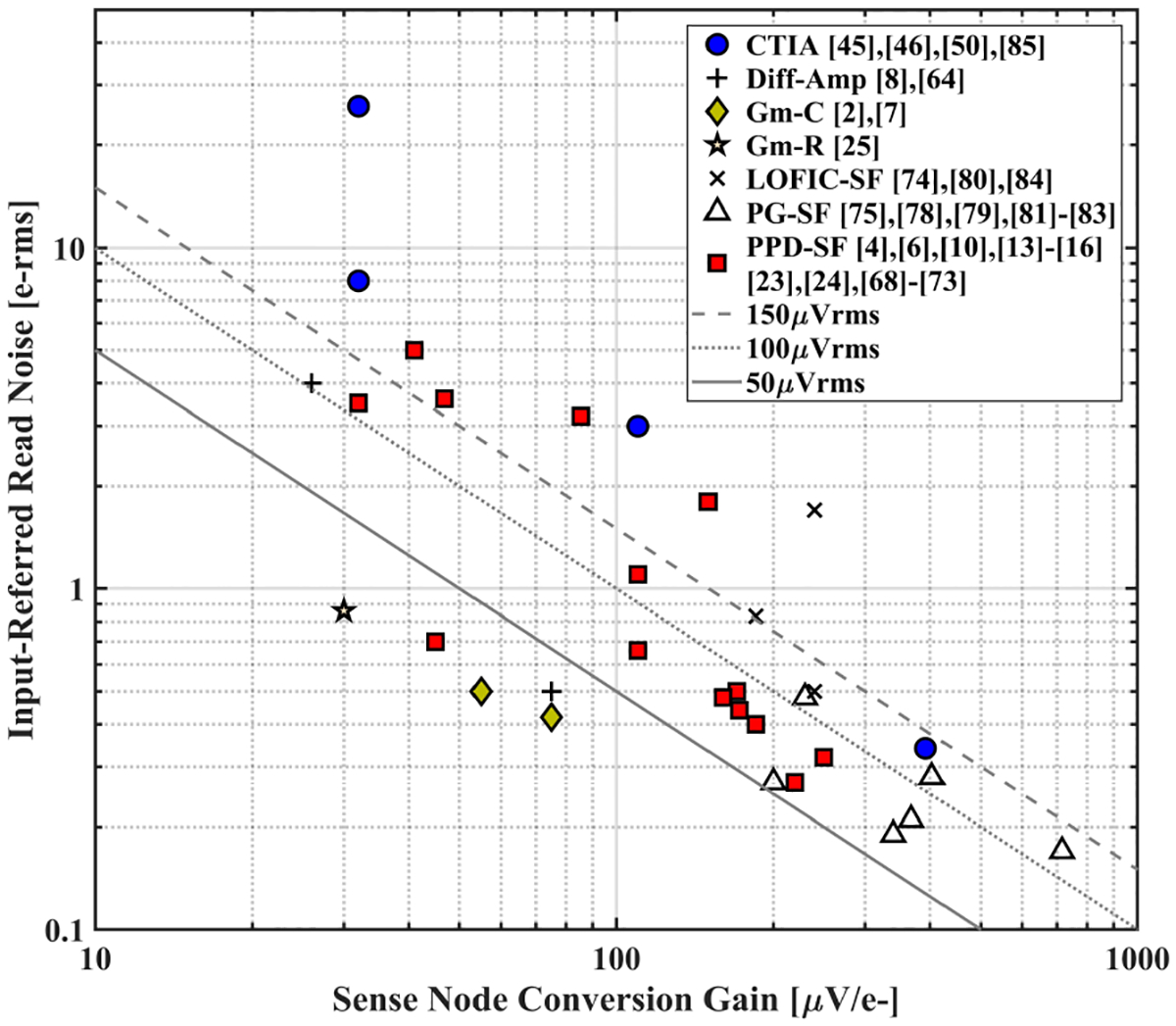
Input-referred read noise and CG of SN performance of reported CIS with IPGAs.

The previous section reported various IPGAs. This section evaluates their performance. Fig. 20 illustrates the read noise of the reported IPGAs along with some PSFs, including state-of-the-art pixels [4], [6], [10], [13], [14], [15], [16], [23], [24], [68], [69], [70], [71], [72], [73], [74] versus the CG or the SN (CG_SN_).

Deng and Fossum [75] reported the lowest input-referred read noise, achieving 0.17e_rms_, which is considered the smallest input-referred noise recorded for CIS sensors to date. In contrast, the state-of-the-art input-referred voltage noise performance is attributed to various amplifier topologies, including the Gm-C amplifier implemented by Ge and Theuwissen [2], and Han and Theuwissen [7], the Gm-R topology by Lotto et al. [25], the shared differential amplifier by Sato et al. [8], and the buried in-pixel SF by Chen et al. [10].

While PSFs can achieve the lowest input-referred charge read noise, they require either a reduction in the SN's diffusion capacitance—leading to a lower pixel full-well capacity (FWC)—or the adoption of specialized technologies, such as front trench deep isolation and pixel sharing to enhance CG without sacrificing FWC [76], [77]. Another approach involves tuning the bulk of the PMOS, although this can introduce design complexity [9], [15].

Conversely, CTIA is well suited for applications, such as fluorescence imaging [46], where noise performance is prioritized over pixel pitch. In high-resolution CIS, where speed and area efficiency are crucial, PSFs remain the preferred choice due to their superior performance compared to other architectures.

Although Gm-C amplifiers offer the lowest input-referred voltage noise, their long integration time can limit their use in large-format CIS. An alternative is using a differential amplifier in an open-loop configuration [8]. However, for small pixel pitches, stacking is often required, where the pixel array is implemented on one wafer, the readout electronics on another, and both are interconnected via Cu–Cu bonding.

**FIGURE 21.**
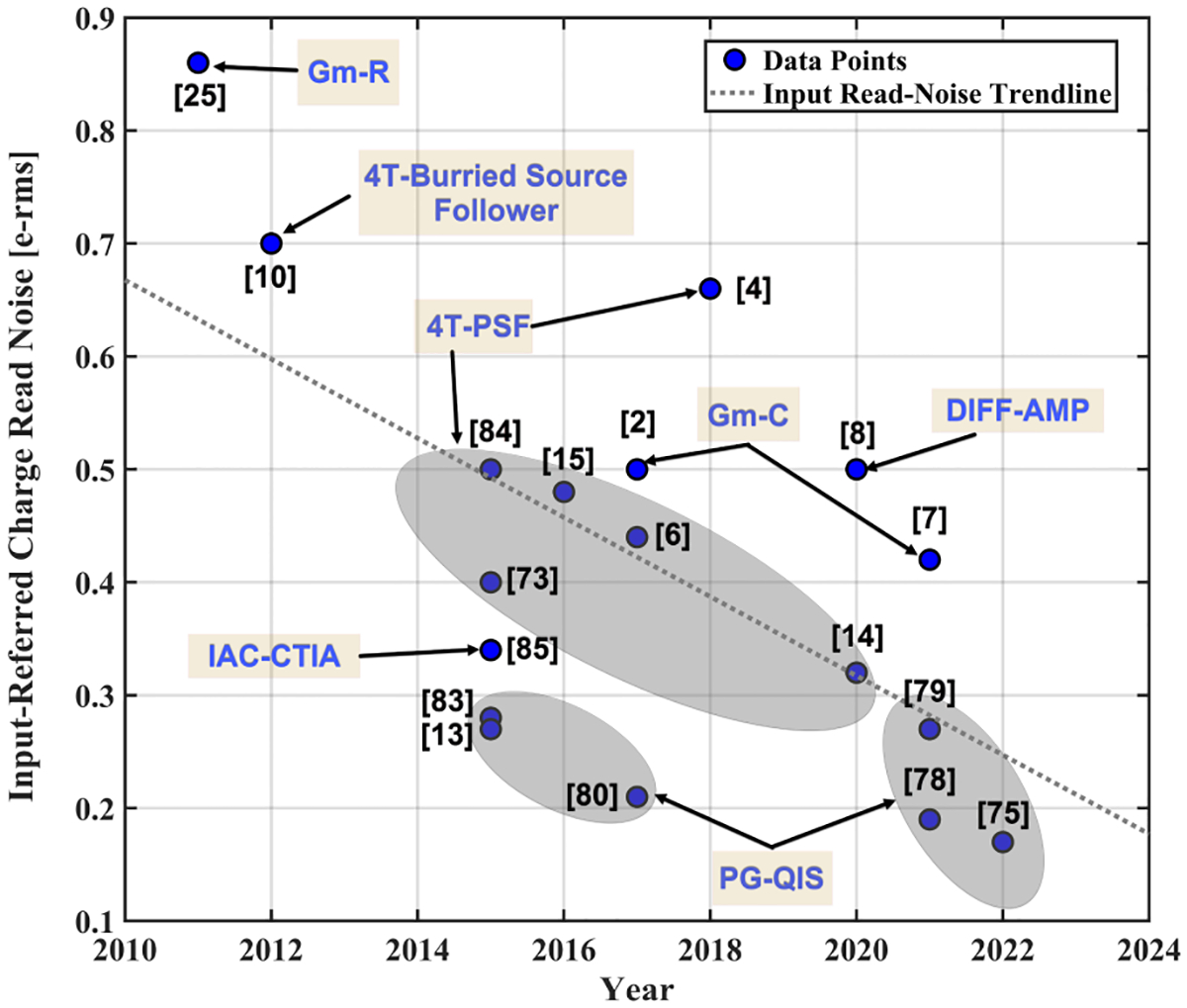
Input-referred read noise trend over the past 15 years.

Fig. 21 presents the trend of input-referred charge read noise over the past 15 years [2], [4], [6], [7], [8], [10], [13], [14], [15], [25], [70], [73], [75], [78], [78], [80], [81], [82], [83], [84], [85], considering only sensors with noise levels below $1e_{\text{rms}}$. A trend line is included to illustrate the gradual decline in read noise over time.

**FIGURE 22.**
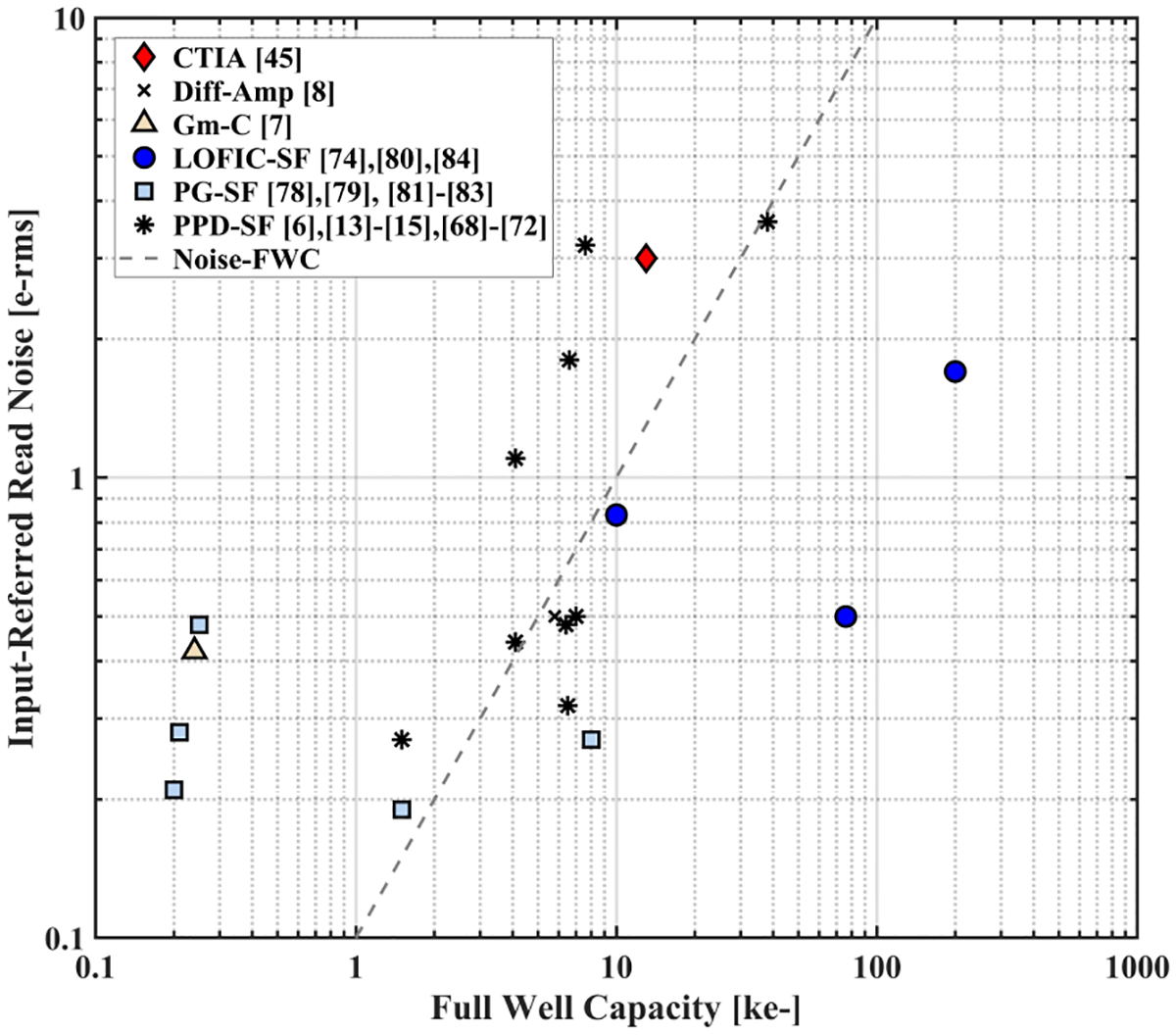
Relationship between FWC and the input-referred read noise of various CIS.

Fig. 22 illustrates the relationship between input-referred read noise and pixel FWC. A conceptual tradeoff line is included to highlight the balance between large-FWC sensors and those with lower read noise. The tradeoff line assumes that a one-decade reduction in input read noise corresponds to a one-decade decrease in FWC. Notably, lateral overflow integrated capacitor (LOFIC) pixels achieve the largest FWC due to their inherent design characteristics. Meanwhile, Ma et al. [78] reported the lowest read noise in quanta image sensors (QIS), with an FWC of 1500e-.

Logarithmic-scale pixel gain sensors (log-scale PGS) are designed to handle a broad dynamic range by compressing the intensity levels of captured light into a logarithmic scale. This approach allows for preserving details in extremely bright and dark regions within a single image, making them particularly suitable for low-light imaging scenarios. In astrophotography, capturing celestial objects often involves dealing with vast differences in brightness, from faint stars to luminous planets [86]. Traditional imaging sensors may struggle to accurately represent such a range without overexposing or underexposing parts of the image. Log-scale PGS can compress this dynamic range, enabling the capture of detailed images of both dim and bright astronomical features simultaneously. This capability enhances the quality of astrophotographs by reducing the need for multiple exposures and extensive postprocessing. An in-pixel logarithmic amplifier can be employed to compress the sensor input [53], thereby extending its dynamic range as discussed in Section II. Alternatively, the PSF associated with LOFIC enables readings in both low and high dynamic range, depending on the lighting conditions [87], [88], [89], which may be useful in astrophotography.

Table 1 compares the noise suppression mechanisms, gain linearity, dynamic range, and CG characteristics of PSF and various IPGA topologies, including Gm-C, Gm-R, CTIA, Log-Amp, and Diff-Amp configurations. The analysis highlights tradeoffs between noise performance, linearity, and dynamic range, which are critical for optimizing CIS design in applications requiring high sensitivity and wide input signal ranges.

Key findings suggest that while PSF offers better gain linearity, IPGA topologies provide programmable gains and noise suppression at the cost of increased design complexity.

## IV. FUTURE TRENDS AND EMERGING TECHNOLOGIES FOR IPGAS

An emerging trend in IPGA design is the development of adaptive architectures that dynamically adjust their gain based on real-time illumination conditions [90]. Such designs can improve sensor performance across a wide range of lighting environments, particularly in scenarios with high dynamic range or rapidly changing scenes. Adaptive IPGA designs may utilize feedback from the pixel or image-level illumination estimates to modulate gain levels, enabling more efficient signal amplification and reducing the risk of saturation or underexposure. Future research could explore robust control schemes and compact circuit implementations that preserve pixel fill factor while offering real-time adaptability.

Recent advancements in machine learning (ML) have opened the door for intelligent noise correction and gain optimization directly on the sensor or at the edge [91], [92], [93]. ML models, such as lightweight convolutional neural networks or reinforcement learning agents, can be trained to optimize gain settings based on scene content or noise statistics. These adaptive algorithms can operate in real time, improving image quality in challenging low-light conditions by compensating for fixed pattern noise and temporal noise and optimizing gain across varying pixel responses. Future work may focus on hardware-efficient implementations of these ML models, cointegrated with sensor readout chains, to enable smart and context-aware image sensing.

## V. CONCLUSION

The various IPGA topologies and associated readout circuits were examined in this article. This review's objective was to classify and demonstrate the non-SF IPGAs utilized in CIS. The use of the CS amplifier topology was avoided in the CIS due to its gain nonlinearity and input bias and range variability, which imposed significant limitations on its application as an IPGA. Various methods to get around those restrictions were proposed and presented in the literature in the past. Using the CSA as a $G_{m}$ cell with a self-reset biasing scheme with a column-shared capacitance or resistor load achieved deep subelectron noise. The fixed gain of the IPGA constrains the dynamic range of the CIS. Hence, a variable CCG can be implemented using the $G_{m}\text{-C}$ IPGA, regulated by the charging time of the column-shared capacitor. For high dynamic range CIS applications, the logarithmic IPGAs provide the best solution. A high dynamic range pixel can be achieved by leveraging the logarithmic relationship between the drain current and the gate-to-source voltage of a mosfet working in the subthreshold region. The unity-gain differential amplifier offers improved gain and linearity compared to PSF-based sensors. Utilizing the ACS technique allows the differential amplifier to be distributed between the pixel and the shared column circuitry. The use of a non-SF gain configuration in a negative capacitive feedback results in a CTIA topology. The CTIA open-loop amplifier can be implemented as either a CSA or a differential amplifier. In conclusion, high-gain IPGAs provide higher CCG compared to the PSFs. They can be used to reduce the effect of column circuit noise on the pixel SN. While the non-SF pixel operation is not straightforward and might need extra area and power consumption, they achieve superior noise performance compared to most PSFs. The integration of ML enables intelligent noise correction and gain optimization in IPGA designs. In addition, it facilitates adaptive adjustment of IPGA gain according to the illumination conditions of the scene being imaged.

## Figures and Tables

**TABLE 1:** Comparison Between PSF and Different IPGA Topologies

TOPOLOGY	NOISE SUPPRESSION	GAIN-LINEARITY	DYNAMIC RANGE	CONVERSION GAIN
**PSF** [15]	PSF adds noise to the SN, CDS, CMS, and different techniques have to be used to suppress the noise	The gain has better overall linearity compared to all IPGAs except the unity-gain diff-amp	Dynamic range is moderate; however, it can be increased by using LOFIC technique	Conversion gain is considered to be low however if pump-gate pixel technology is used it could be increased
**Gm-C** [3]	Noise is suppressed by increasing the charging time of the column sampling capacitor	The gain depends on the bias point of the Gm transistor which may degrade with PVT variations	A programmable dynamic range can be achieved by controlling the charging time leading to a tradeoff between noise performance and dynamic range	Programmable conversion gain can be achieved by changing the charging time
**Gm-R** [25]	Noise is suppressed by the open-loop voltage gain of the Gm-R amplifier	The gain is bias-dependent and degrades with PVT variations	Dynamic range is limited by the output range of the in-pixel amplifier	Fixed high conversion gain can be achieved, which limits the dynamic range of the Gm-R topology
**CTIA** [46]	Noise suppression is dependent on how small the feedback capacitor can be implemented	The gain of the CTIA becomes more linear as the open-loop gain of the amplifier approaches infinity	A tradeoff between noise performance and dynamic range occurs	Low conversion gains are achieved due to the usually large required dynamic range
**Log-Amp** [53]	The transconductance of the CS amplifier inside the log-amp must be maximized to ensure noise suppression	The Gain is linear over the wide input range	Wide dynamic range can be easily achieved using this topology	Conversion gain isn’t the key characteristic of log-amps and hence is considered to be relatively small
**Diff-Amp** [8]	When used in a non-unity configuration noise is suppressed by the voltage gain of the configuration	When used in a unity gain feedback, it is considered the most linear in-pixel amplifier in terms of gain	Dynamic range is dependent on the configuration of the diff-amp, but is considered generally moderate	Considered to have large fixed conversion gains when compared to other structures
